# Application of machine learning and deep learning in metabolic dysfunction-associated steatotic liver disease: a systematic review and meta-analysis

**DOI:** 10.1016/j.jare.2025.08.042

**Published:** 2025-08-22

**Authors:** Huan Zhang, Xiangyu Wu, Wenjing Ni, Jiali Wu, Sisi Zhou, Leyao Jia, Mingyang Jin, Sitian Zhao, Zhenyao Jiang, Chao Wu, Yuxiang Sun, Junping Shi, Jie Li

**Affiliations:** a Department of Infectious Disease, Nanjing Drum Tower Hospital, Affiliated Hospital of Medical School, Nanjing University, Nanjing, Jiangsu, China; b Department of Infectious Diseases, Nanjing Drum Tower Hospital Clinical College of Nanjing University of Chinese Medicine, Nanjing, Jiangsu, China; c Institute of Viruses and Infectious Diseases, Nanjing University, Nanjing, Jiangsu, China; d Department of Infectious Diseases, Nanjing Drum Tower Hospital Clinical College of Nanjing Medical University, Nanjing, Jiangsu, China; e Department of Infectious Diseases, The Affiliated Hospital of Hangzhou Normal University, Hangzhou, Zhejiang, China; f Jiangsu Key Laboratory of Integrated Traditional Chinese and Western Medicine for Prevention and Treatment of Senile Diseases, Institute of Translational Medicine, Medical College, Yangzhou University, Yangzhou, Jiangsu, China

**Keywords:** Artificial intelligence, Machine learning, Deep learning, Diagnostic model, Metabolic dysfunction-associated steatotic liver disease, Metabolic dysfunction-associated steatohepatitis, Liver fibrosis

## Abstract

**Introduction:**

Metabolic dysfunction-associated steatotic liver disease (MASLD) can progress to metabolic dysfunction-associated steatohepatitis (MASH) and liver fibrosis, contributing to a heavier global health burden. Non-invasive diagnostic tools developed using machine learning (ML) and deep learning (DL), two representative artificial intelligence algorithms, are increasingly being explored for MASH and its related fibrosis assessment.

**Objectives:**

This study aimed to compare the diagnostic performance of different ML and DL models and identify the top-performing models for diagnosing MASH and associated liver fibrosis.

**Methods:**

A systematic review and meta-analysis were conducted across PubMed, Web of Science, Embase and Cochrane Library from inception to May 18, 2025. Pooled area under the receiver operator characteristic curve (AUROC) values with 95 % confidence interval (CI) were calculated. Accuracy, specificity, sensitivity, positive predictive values, and negative predictive values were also recorded.

**Results:**

Of 4,314 studies initially identified, 106 met the inclusion criteria, with 35 studies (ML: n = 28; DL: n = 7) providing data for analysis. Logistic Regression and Neural Network are the most commonly algorithms applied in ML and DL, respectively. The pooled AUROCs for diagnosing MASH were 0.833 (95 %CI: 0.806–0.860) for ML models and 0.841 (95 %CI: 0.782–0.900) for DL models. Light Gradient Boosting Machine (LightGBM) and ResNet50 were the best-performing models for diagnosing MASH within ML and DL algorithms, respectively, achieving corresponding AUROCs of 0.920 (95 %CI: 0.916–0.924) and 0.960 (95 %CI: 0.951–0.969). For fibrosis diagnosis, ML models had a pooled AUROC of 0.826 (95 %CI: 0.792–0.860), with Categorical Boosting (CatBoost) achieving the highest AUROC of 0.960 (95 %CI: 0.950–0.970). DL models yielded the pooled AUROC of 0.875 (95 %CI: 0.816–0.934) for fibrosis diagnosis.

**Conclusions:**

Both ML and DL models demonstrated strong diagnostic performance for MASH and liver fibrosis, with DL achieving marginally higher AUROCs. AI-driven approaches show promise in MASLD management.

## Highlights

• Machine learning and deep learning perform well in diagnosing MASH and its related liver fibrosis.
• Logistic Regression and Neural Network algorithms are the most commonly used tools.
• Machine learning had AUROC of above 0.8 in MASH and fibrosis diagnosis, peaking at 0.9 for cirrhosis.
• Deep learning had pooled AUROCs higher than 0.8 for diagnosing MASH and fibrosis.
• Artificial intelligence-based models are promising approaches for MASH and fibrosis diagnosis.

## Graphical abstract

**Figure:**
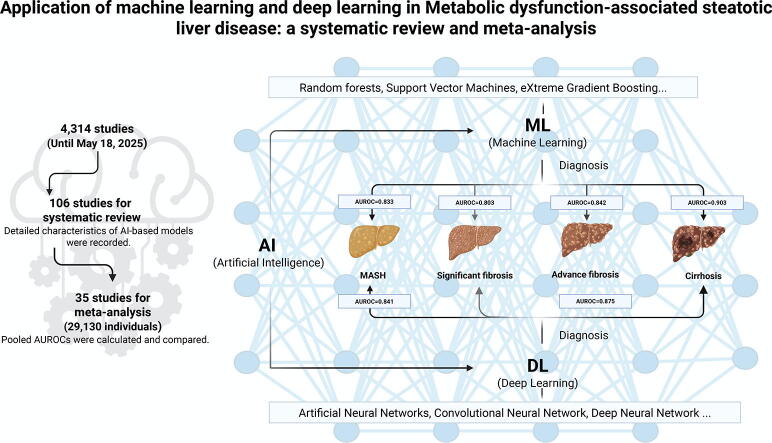

## Introduction

Metabolic dysfunction-associated steatotic liver disease (MASLD), formerly known as nonalcoholic fatty liver disease (NAFLD) and metabolic dysfunction-associated fatty liver disease (MAFLD), is one of the most prevalent causes of chronic liver disease worldwide [1]. With the rising incidence of obesity and diabetes, the prevalence of MASLD has approached 30 % and continues to increase, highlighting its emergence as a significant global public health concern [[2], [3], [4]]. It is estimated that approximately 25 % of individuals with MASLD will progress to metabolic dysfunction-associated steatohepatitis (MASH) and liver fibrosis, which serve as key predictors of MASLD progression [5], contributing to an elevated risk of cirrhosis, hepatocellular carcinoma (HCC), liver transplantation, and increasing liver-related mortality [[6], [7], [8]]. Therefore, accurate diagnosis and prediction of MASH and its related liver fibrosis are crucial for early intervention.

Currently, liver biopsy remains the gold standard for diagnosing MASH and its associated fibrosis. However, due to its invasiveness and high cost, it is not recommended for routine screening in the MASLD population. Additionally, diagnostic variability exists among different pathologists, leading to inconsistencies in interpretation [9]. Non-invasive tests (NITs) based on simple or classic regression methods, such as fibrosis-4 index (FIB-4), aspartate aminotransferase-platelet ratio (APRI), and NAFLD fibrosis score (NFS), have shown limited robustness across different study populations [10].

With the advancement of artificial intelligence (AI), machine learning (ML) has demonstrated significant advantages in image processing and diagnostic problem-solving, making it a promising tool in the medical field. AI involves the use of algorithms and models that enable machines to perform tasks requiring human-like intelligence. ML, a subset of AI, consists of algorithms that iteratively learn patterns from input data without explicit programming [11]. Commonly, ML can be grouped into ensemble and non-ensemble methods. Ensemble methods improve diagnostic or predictive performance by combining multiple ML models, with prominent examples including eXtreme Gradient Boosting (XGBoost), Random forests (RF), Adaboost Adaptive Boosting (AdaBoost), Light Gradient Boosting Machine (LightGBM), Gradient Boosting (GB), Categorical Boosting (CatBoost), and Gradient Boosting Machine (GBM) [12]. In contrast, non-ensemble methods operate as standalone models, such as Support Vector Machines (SVM), Logistic Regression (LR), Decision Tree (DT), K-Nearest Neighbor (KNN), Linear Discriminant Analysis (LDA), Naive Bayes (NB), and Classification And Regression Tree (CART) [13]. Additionally, deep learning (DL), a specialized branch of ML, utilizes multi-layered computational models to learn hierarchical data representations. Notable DL architectures include Convolutional Neural Network (CNN) and Recurrent Neural Network (RNN) [14].

ML and DL models have been increasingly utilized for biomarker discovery and predictive modeling to assess the severity and prognosis of MASH and its associated liver fibrosis or cirrhosis [[15], [16], [17], [18]]. Furthermore, ML and DL algorithms can be trained for automated image analysis, including liver histological images [19] and non-invasive images [20], such as computerized tomography scan and magnetic resonance elastography (MRE), achieving diagnostic performance comparable to that of expert clinicians.

Applications of ML and DL have shown promise in enhancing diagnostic, prognostic, and treatment response prediction of MASH and its associated liver fibrosis. However, few systematic reviews and meta-analyses have comprehensively evaluated their performance in this rapidly evolving field. This study aimed to assess the performance of ML and DL in diagnosing MASH and related liver fibrosis and to compare the diagnostic performance between the two approaches.

## Methods

### Search strategy

This meta-analysis included studies that developed ML or DL models for diagnosing or predicting MASH, and MASH-related fibrosis/cirrhosis. A systematic literature search was conducted in PubMed, Web of Science, Embase and Cochrane Library from inception to May 18, 2025. The search terms included “NAFLD”, “MAFLD”, “MASLD”, “MASH”, “NASH”, “fibrosis”, “cirrhosis”, “artificial intelligence”, “AI”, “machine learning”, “ML”, “deep learning”, “DL”, “prognosis”, and “diagnosis”. Full search strategies for each database are provided in supplemental materials. Additionally, the study protocol was registered with the International Prospective Register of Systematic Reviews (PROSPERO, CRD42024619981).

## Inclusion and exclusion criteria

During the screening process, the inclusion and exclusion criteria were as followed to determine which studies would be taken into the analyses. The inclusion criteria: (1) Studies involving patients aged 18 years or older diagnosed with nonalcoholic steatohepatitis (NASH)/MASH; (2) Prospective or retrospective cohort studies, case-control studies, randomized controlled trials and cross-sectional studies; (3) Diagnosis of NASH/MASH and/or related liver fibrosis based on liver biopsy and/or imaging; (4) Developing and/or validating the performance of ML for diagnosing NASH/MASH and/or related liver fibrosis stages, or predicting the risk of liver fibrosis, liver-related adverse events and/or all-cause mortality in NASH/MASH patients. The exclusion criteria: (1) Reviews, abstracts, book chapters, editorials, news and other non-original articles; (2) Studies that did not include human participants; (3) Studies included participants with NASH/MASH combined with other chronic liver diseases (e.g., chronic hepatitis B); (4) Non-English publications; (5) Studies that did not use ML methods or only used simple logistic or linear regression methods.

## Study selection and data extraction

All identified studies were uploaded to Endnote, and duplicate entries were removed. Two independent reviewers (H.Z. and X.W.) screened the titles and abstracts to determine eligibility. Full-text articles of eligible studies were then assessed, and relevant data were extracted independently by the same two reviewers. In cases where there was disagreement regarding an abstract or full-text article, a third independent reviewer (W.N.) was consulted to resolve the conflict and make the final decision. ML and DL models applied in each study with available data were all extracted for analysis. Data extraction followed the CHecklist for critical Appraisal and data extraction for systematic Reviews of prediction Modelling Studies (CHARMS) [21] (Table S1). Studies that met the inclusion criteria but lacked sufficient data for meta-analysis were only included in the systematic review.

## Outcomes

The primary outcome was to compare the diagnostic performance of ML and DL models in detecting MASH and its related liver fibrosis. The secondary outcome was to identify the top-performing models within ML and DL categories respectively for diagnosing MASH and related fibrosis.

## Quality assessment

The quality of the included studies was assessed using the Quality Assessment of Diagnostic Accuracy Studies-2 (QUADAS-2) tool [22]. This tool evaluates diagnostic accuracy, risk of bias, and applicability across four domains: patient selection, index test, reference standard, and flow and timing. The risk of bias for each domain was classified as low, high, or unclear. The QUADAS-2 assessment results were visualized using Cochrane Collaboration Review Manager Software (RevMan, version 5.4).

### Statistical analysis

MedCalc (23.0.5) was used for meta-analysis, and area under the receiver operator characteristic curve (AUROC) was used as the statistic for effect analysis. Cochrane's Q test combined with *I^2^* statistic were used to determine the heterogeneity. *I^2^* > 50 % indicated considerable heterogeneity where a random-effects model was used; otherwise, a fixed-effect model was used.

## Results

### Characteristics of eligible studies

**Fig. 1:**
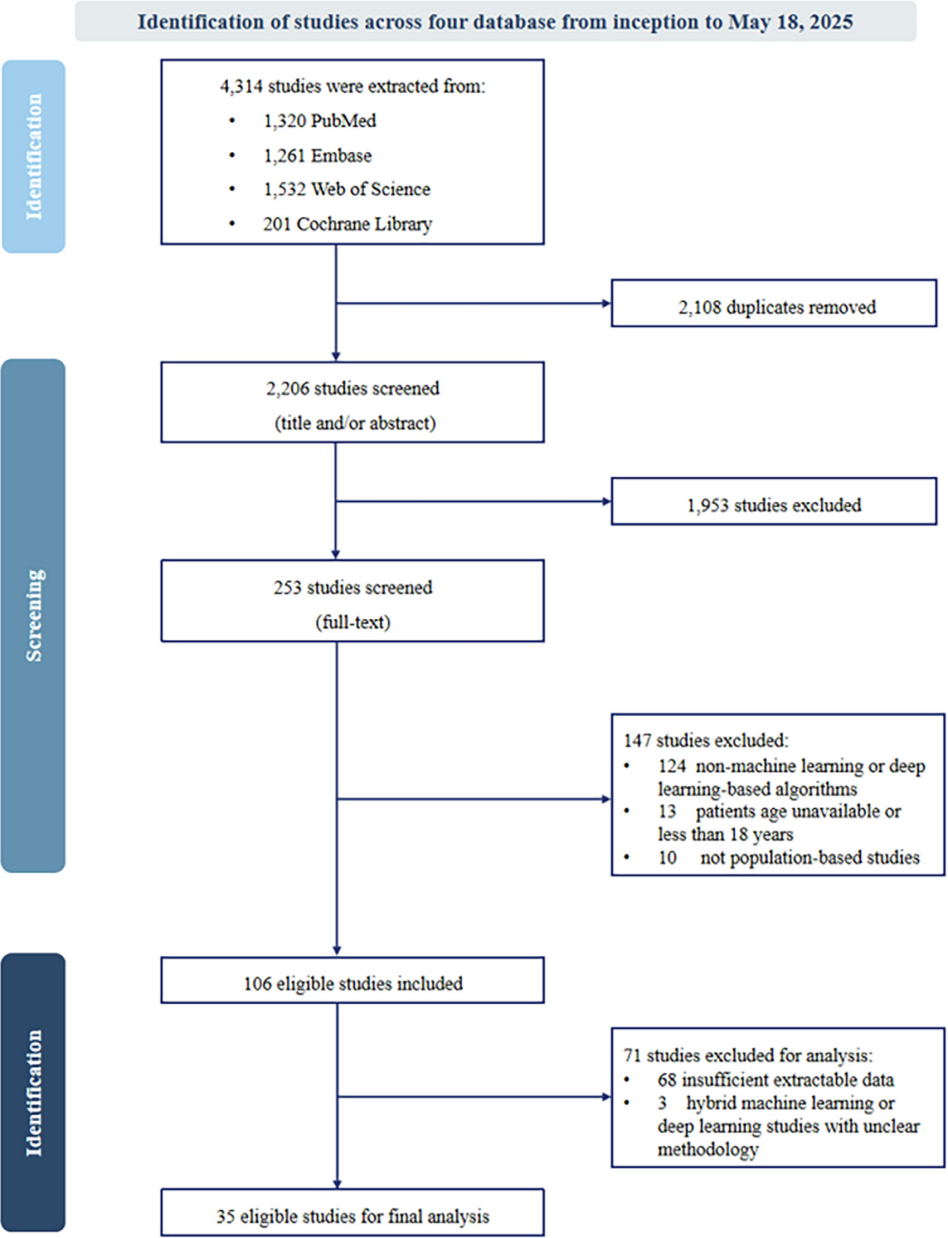
Flow diagram for systematic reviews and meta-analyses.

The process of study selection is illustrated in Fig. 1. A comprehensive search across four databases initially identified 4,314 studies. After removing 2,108 duplicates, 2,206 studies were screened by reviewing titles and abstracts, of which 1,953 did not meet the inclusion criteria. Subsequently, a full-text review was conducted for 253 studies, resulting in the exclusion of 147 studies. A total of 106 studies were eligible according to the inclusion and exclusion criteria.

**Fig. 2:**
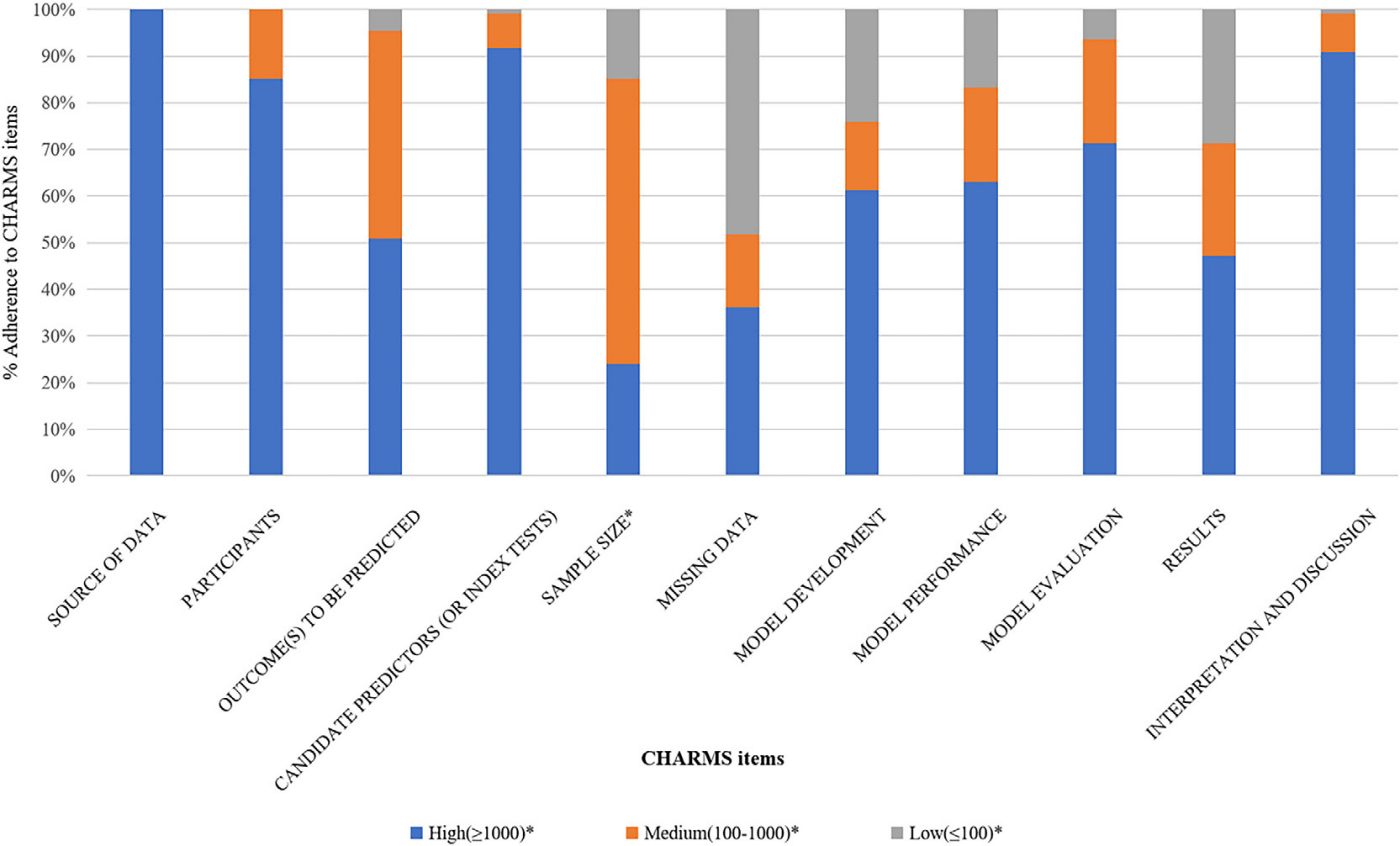
Summary of 106 eligible studies adherence to CHecklist for critical Appraisal and data extraction for systematic Reviews of prediction Modelling Studies (CHARMS) reporting guidelines.

Among the 106 studies, 43 % (46/106) of studies developed for diagnostic models used liver biopsies as the reference standard. Other diagnostic methods included shear wave elastography, MRE, and transient elastography (TE). There are 8 % (9/106) of studies that developed prognostic models to predict liver-related adverse outcomes and all-cause mortality in individuals with MASLD/MASH. The summary of study adherence to CHARMS is presented in Fig. 2.

A column line chart was plotted to delineate the number of studies applying ML and DL-based models in diagnosing MASH and its related fibrosis and predicting mortality among the 106 eligible studies (Fig. S1). A rapid growth was observed after 2021. Out of the 106 studies, 35 studies covering 29,130 individuals with available data were extracted and included for final analysis, of which 28 and 7 studies employed ML and DL, respectively. Among traditional ML algorithms, LR was the most commonly applied algorithm, followed by RF, SVM, and XGBoost. For DL-based models, Neural Network (NN) algorithms, including Artificial Neural Networks (ANN), CNN, Deep Neural Network (DNN), were the most frequently employed models.

As summarized in Table 1, Table 2, the common predictors used in ML and DL models within 35 studies included demographic characteristics (age, gender, body mass index, etc.), laboratory parameters (aminotransferase levels, albumin, glycated hemoglobin, etc.), and clinical factors (blood pressure, the presence of type 2 diabetes mellitus, etc.). In addition, some researchers integrated omics-based features to develop models, such as radiomics, urinary steroid metabolome, and fecal metaproteomics [[23], [24], [25]]. The full information of ML and DL models for 106 studies was detailed in Table S2 and Table S3, respectively.

**Table 1:** Characteristics of the 28 eligible studies with available data for machine learning model analysis.

Study	Outcomes	Study country/area	Algorithms^△^	Participants (No.)	Training size (No.)	Testing size (No.)	Validation size (No.)	Used features/Supplementary information
Canbay et al 2019 [48]	Fibrosis	NR	LR	286	164	NR	122 (Internal validation)	Age, GGT, HbA1c, adiponectin, and M30
Eslam et al 2016 [49]	Fibrosis	Multiple countries	Decision Tree	4,277	1,992	NR	2,285 (External validation)	Interferon-k4 gene-IFNL genotype, age, gender and routinely assessed clinical and laboratory variables
Verma et al 2024 [33]	Significant fibrosis	14 centres in 8 Asia countries	RF	1,656	1,656	NR	NR	Age, aminotransferase levels, PLT, fasting plasma glucose, diabetes control, and GGT.
Sowa et al 2013 [26]	Fibrosis	Germany	RF	126	126	NR	NR	ALT, AST, M30, M60, HA
Chen et al 2021 [50]	Fibrosis	NR	LR	22	22	NR	NR	Radiomics features of 18F-FDG PET images
Fan et al 2024 [51]	Fibrosis	China, United States	LR	946	946	NR	NR	LSM, age, sex, PLT, albumin, total bilirubin
Feng et al 2021 [52]	Fibrosis	China	RF	553	553	NR	275 (Internal validation)	BMI, pro-collagen type III, collagen type IV, AST, A/G ratio
Moolla et al 2020 [24]	Fibrosis	NR	GMLVQ	275	275	NR	NR	Urinary steroid metabolome
Wang et al 2019 [53]	Fibrosis	China	ML*	344	206	138	NR	/
Bastati et al 2023 [54]	MASH	NR	RF-based on UDC	76	46	NR	30 (Internal validation)	TBil, ALT, AST, ALP, GGT, TG, HDL-C, glucose
Chang et al 2023 [34]	Fibrosis/ MASH with significant fibrosis	California, Texas, and Arizona	RF	1,370	1,370	NR	NR	Gender, age, BMI, ALP, TBil, ALT, AST, albumin, WBC, PLT, HbA1c, TC, LDL, HDL-C, TG, T2DM, and hypertension status
Wu et al 2022 [55]	MASH/ Fibrosis	USA	GB	492	492	NR	NR	AST, ALT and TG are important risk factors for NASH. AST, HbA1c and HDL-C are important variables in predicting advanced fibrosis.
Njei et al 2024 [37]	MASH	USA	XGBoost	5,156	1,719	1,718	1,719 (Internal validation)	ALT, GGT, Platelet count, Waist circumference, Age
Dabbah et al 2025 [56]	Fibrosis	Israel	XGBoost	1,158	618	NR	540 (Internal validation)	Age, sex, BMI, DM/impaired fasting glucose, HbA1c, hypertension, AST, ALT, GGT, ALP, platelets, albumin, total cholesterol, LDL-c, HDL-C, TG
Feng et al 2024 [57]	Fibrosis	China	LR	571	399	NR	172 (External validation)	αC18:3, γ-C18:3, sex, age, BMI, SBP, duration of diabetes, Triglyceride glucose index, HDL-C, HbA1c, fasting c-peptide, and c-peptide 2 h postprandial
Mouskeftara et al 2024 [38]	MASH	Greece	XGBoost	37	37	NR	NR	HOMA-IR, BMI, platelets count, LDL-c, ferritin, AST, FA 12:0, FA 18:3 ω3, FA 20:4 ω6/FA 20:5 ω3, CAR 4:0, LPC 20:4, LPC O-16:1, LPE 18:0, DG 18:1_18:2, and CE 20:4
Snethlage et al 2024 [58]	Fibrosis	Netherlands	Extra-Trees classification	453	453	NR	NR	Type 1 diabetes duration, age, sex, BMI, stimulated C-peptide/creatinine ratio, SBP, daily insulin dose, time below range/time in range/time above range of glucose, and HbA1c
Zamanian et al 2024 [59]	MASH	Japan	RF	176	176	NR	NR	BMI, ALT, TC,HDL-C, Ezetimibe, lipoprotein level Lp(a), Loge(Lp(a)), TG, Creatinine, HbA1c, Fibrate, and Sex
Yan et al 2024 [29]	MASH	China	XGBoost	587	406	NR	181 (External validation)	Neutrophil percentage, AST/ALT ratio, hematocrit, creatinine, uric acid, prealbumin
Huang et alv2025 [60]	MASH/ mortality	China, Finland	RF	118,182	117,877	NR	305 (External validation)	BMI, AST, Tyrosine, phospholipid-to-total lipid ratio in VLDL
Liu et al 2025 [39]	MASH/ Fibrosis	China	XGBoost	561	456	NR	105 (External validation)	AST: ALT ratio, homeostatic model assessment, fibrotic non-alcoholic steatohepatitis index, BMI, diabetes, white blood cell, neutrophil, basophil, PLT, APTT, fibrinogen, insulin, ALT, AST, GGT, HbA1c
Alkhouri et al 2025 [61]	Fibrosis	USA, Hong Kong, France, Australia, India, etc.	RF, gradient boosting machines, and XGBoost	3,630	827	1,504	1,299 (External validation)	Age, sex, BMI, serological parameters (AST, ALT, platelet, GGT, etc.), LSM assessed by VCTE.
Panagiotopoulos et al 2025 [62]	MASH/ Fibrosis	USA	Bayesian Information Criterion-based stepwise logistic regression	140	140	NR	NR	PDFF, AST, and sex (MASH); PDFF and MRE (Fibrosis)
Stefanakis et al 2025 [40]	MASH and F2-F3	USA, Italy, Australia, Greece	Categorical Boosting	443	353	NR	90 (Internal validation)	ALT, AST, BMI, metabolic syndrome components, 3-ureidopropionate, alpha-ketoglutarate
Wakabayashi et al 2025 [63]	Fibrosis	Japan	Support Vector Machine	463	370	93	NR	Age, sex, BMI, diabetes mellitus, hypertension, hyperlipidemia, AST, ALT, GGT, HbA1c, TG, HDL-C, LDL-C, FBG.
Xiong et al 2025 [64]	Fibrosis	China	XGBoost	746	522	NR	224 (Internal validation)	TG, ALB, INR, HDL-C
Calès et al 2025 [42]	Fibrosis	France, Switzerland	ADORE software	1,051	637	NR	414 (Internal validation)	Aspartate aminotransferase, alanine aminotransferase, gamma-glutamyltransferase, ALP, bilirubin, albumin, platelets, prothrombin index [%, or international normalized ratio] and urea, all adjusted on age, weight, height and diabetes,hyaluronate and alpha2-macroglobulin, LSM
Jamialahmadi et al 2025 [65]	Fibrosis	Iran	LR	512	358	NR	154 (Internal validation)	Hemoglobin, FBG, Skeletal Muscle Mass, ALT, Triglycerides, AST.

Note: ^△^ indicated that if studies employed various models, only diagnostic metrics of the best-performing models within this study were displayed in this table. * indicated that studies did not specify the exact machine learning algorithms used; these cases are labeled as ML in this table. Abbreviations: CHAID, Chi-squared Automatic Interaction Detector; GB, Gradient Boosting; GBM, Gradient Boosting Machine; GMLVQ, Generalized Matrix Learning Vector Quantization; KNN, K-Nearest Neighbor; LR, Logistic Regression; ML, machine learning; NR, Not reported; RF, Random forests; XGBoost, eXtreme Gradient Boosting; NASH, non-alcoholic steatohepatitis; DM, diabetes mellitus; T2DM, type 2 diabetes mellitus; INR, International normalized ratio; LSM, liver stiffness measurement; VCTE, vibration-controlled transient elastography; ALT, alanine aminotransferase; AST, aspartate aminotransferase; ALP, alkaline phosphatase; Tbil, total bilirubin; ALB, albumin; GGT, gamma-glutamyl transferase; HbA1c, glycated hemoglobin; HOMA-IR: Homeostasis Model Assessment of Insulin Resistance; TG, triglyceride; HDL-C, high-density lipoprotein cholesterol; LDL-C, low-density lipoprotein cholesterol; FBG, fasting blood glucose; BMI, body mass index; PDFF, proton density fat fraction; SBP, systolic blood pressure; WBC, white blood count; PLT, platelet count; HA, hyaluronic acid.

**Table 2:** Characteristics of the 7 eligible studies with available data for deep learning model analysis.

**Study**	**Outcomes**	**Study country/area**	**Algorithm^△^**	**Participants** **(No.)**	**Training size (No.)**	**Testing size (No.)**	**Validation size (No.)**	**Used features/ Supplementary information**
Cunha et al 2022 [20]	Fibrosis	USA	CNN	756	675	81	NR	CNN-based MRE stiffness measurements
Marti-Aguado et al 2021 [35]	MASH	Spain	MATLAB software	156	156	NR	NR	/
Gao et al 2023 [66]	MASH	China	FCNN	261	209	NR	52 (Internal validation)	Surface enhanced Raman spectroscopy
Li et al 2023 [31]	MASH	China	DNN	766	613	NR	153 (Internal validation)	Age, sex, prior hypertension, prior diabetes, six body composition (namely arm circumference, percent body fat, bone mineral content, basal metabolic rate, body cell mass and visceral fat area)
Okanoue et al 2021 [67]	MASH	Japan	NN	398	324	NR	74 (External validation)	Age, sex, height, weight, waist circumference, AST, ALT, gGGT, cholesterol, TG, and PLT
Okanoue et al 2021 [68]	MASH	Japan	NN	434	324	NR	110 (External validation)	Age, sex, height, weight, waist circumference, AST, ALT, GGT, cholesterol, triglyceride, platelet count, and type 4 collagen 7 s
Chattopadhyay et al 2025 [69]	MASH	China	CNN	137	110	27	NR	Ultrasound B-scan image features

Note: ^△^ indicated that if studies employed various models, only diagnostic metrics of the best-performing models within this study were displayed in this table. Abbreviations: A/G, albumin/globulin; ALT, alanine aminotransferase; ALP, alkaline phosphatase; ANN, Artificial Neural Network; AST, aspartate aminotransferase; BMI, body mass index; CAE, Convolutional Auto Encoder; CNN, Convolutional Neural Network; Cre, creatinine; CRP, C-reactive protein; DBP, diastolic blood pressure; DL, deep learning; DNN, Deep Neural Network; GAN, Generative Adversarial Networks; GGT, gamma glutaryl transferase; GPT, glutamic pyruvic transaminase; HA, hyaluronic acid; HbA1c, glycated hemoglobin; HDL-C, high density lipoprotein cholesterol; LDL, low-density lipoprotein; Lp(a), Lipoprotein(a); NN, Neural Network; PLT, platelets; PT/INR, prothrombin time test/International Standardized Ratio; T2DM, type 2 diabetes mellitus; Tbil, total bilirubin; TC, total cholesterol; TG, triglyceride; UA, uric Acid; WC, waist circumference; WBC, white blood count.

## Quality assessment

The QUADAS-2 tool identified 10 studies as having a high or unclear risk of bias in patient selection, primarily due to non-consecutive or non-random patient inclusion [26]. Five studies showed potential bias in the flow and timing domain, as there was a long interval between the diagnostic tests and the reference liver biopsy. Additionally, eight studies had an unclear risk of bias in the index test domain due to the lack of clear pre-specification of thresholds. The reference standard domain was generally low risk, as the majority of studies (31/35) used liver biopsy as the reference standard. Overall, all 35 studies were considered to have good applicability. Detailed assessments are presented in Fig. S2.

## Performance of ML and DL-based models in diagnosing MASH

**Fig. 3:**
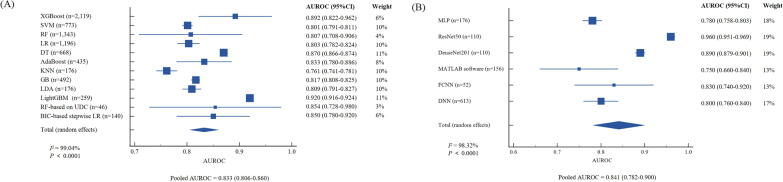
**Forest plots of AUROCs for diagnosing MASH. (A) Machine learning models; (B) Deep learning models.** Abbreviations: AUROC, area under the receiver operator characteristic curve; CI: confidence interval; MASH, metabolic dysfunction-associated steatohepatitis; AdaBoost, Adaboost Adaptive Boosting; BIC, Bayesian Information Criterion; DT, Decision Tree; GB, Gradient Boosting; GBM, Gradient Boosting Machine; KNN, K-Nearest Neighbor; LR, Logistic Regression; RF, Random forests; SVM, Support Vector Machines; UDC, Unsupervised Deep Clustering; XGBoost, eXtreme Gradient Boosting.

A total of 14 studies with 9,308 individuals developed models for diagnosing MASH, with 10 studies covering 12 ML-based models and 5 studies inclusive of 6 DL-based models included for analysis. The overall AUROCs for ML and DL models were 0.834 (95 %CI: 0.810–0.859). Pooled AUROCs were 0.833 (95 %CI: 0.806–0.860) for ML models and 0.841 (95 %CI: 0.782–0.900) for DL models without significant heterogeneity (*I^2^* = 0.00 %, *P* = 0.810) (Fig. S3). Among ML models, LightGBM achieved the highest AUROC of 0.920 (95 %CI: 0.916–0.924), followed by XGBoost with an AUROC of 0.892 (95 %CI: 0.822–0.962). Most ML models demonstrated AUROCs higher than 0.8, except for KNN, which reached 0.761 (95 %CI: 0.741–0.781) (Fig. 3A). As for DL models, ResNet50 showed the best performance with an AUROC of 0.960 (95 %CI: 0.951–0.969), followed by DenseNet201 yielding an AUROC of 0.890 (95 %CI: 0.879–0.901) (Fig. 3B). Both ML and DL studies demonstrated substantial heterogeneity (ML: *I*^^*2*^^ = 99.04 %; DL: *I*^^*2*^^ = 98.32 %, both *P* < 0.0001; Fig. 3), as indicated by Cochran's Q test. Detailed diagnostic performance metrics, including accuracy, specificity, sensitivity, positive predictive values (PPV), and negative predictive values (NPV), are provided in Table S4.

## Performance of ML and DL-based models in diagnosing MASH-related fibrosis

**Fig. 4:**
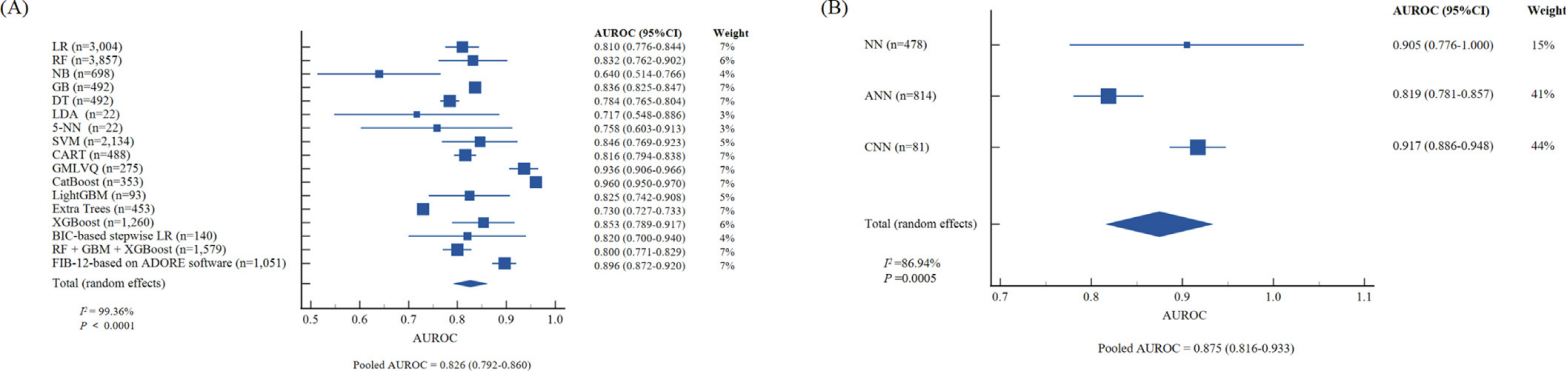
**Forest plots of AUROCs for diagnosing liver fibrosis. (A) Machine learning models; (B) Deep learning models.** Abbreviations: AUROC, area under the receiver operator characteristic curve; CI: confidence interval; BIC, Bayesian Information Criterion; CatBoost, Categorical Boosting; CART, Classification And Regression Tree; CNN, Convolutional Neural Network; DT, Decision Tree; GB, Gradient Boosting; GBM, Gradient Boosting Machine; GMLVQ, Generalised Matrix Learning Vector Quantisation; LDA, Linear Discriminant Analysis; LR, Logistic Regression; NB, Naive Bayes; RF, Random forests; SVM, Support Vector Machines; XGBoost, eXtreme Gradient Boosting; 5-NN, 5-Nearest Neighbor.

A total of 24 studies incorporating 20,985 individuals were recruited to develop models for diagnosing MASH-related fibrosis. Among them, 21 studies used ML models, and 5 studies employed DL models. The pooled AUROC for combined ML and DL models was 0.839 (95 %CI: 0.809–0.868). Individually, ML models achieved a pooled AUROC of 0.826 (95 %CI: 0.792–0.860), while DL models showed superior performance with an AUROC of 0.875 (95 %CI: 0.816–0.934), both without significant heterogeneity (*I^2^* = 49.86 %, *P* = 0.158) (Fig. S4). Specifically, when comparing the 17 different ML-based models, CatBoost showed the highest AUROC of 0.960 (95 %CI: 0.950–0.970), with Generalised Matrix Learning Vector Quantisation (GMLVQ) coming in second at an AUROC of 0.936 (95 %CI: 0.906–0.966) (Fig. 4A). However, NB showed limited diagnostic capability, with an AUROC of 0.640 (95 %CI: 0.514–0.766). For DL models, only NN, CNN, and ANN were employed for diagnosing MASH-related fibrosis, with CNN reaching the best AUROC of 0.917 (95 %CI: 0.886–0.948) (Fig. 4B). NN and ANN also had sound performance, with an AUROC of 0.905 (95 %CI: 0.776–1.000) and 0.819 (95 %CI: 0.781–0.857), respectively. ML and DL studies demonstrated substantial heterogeneity (ML: *I*^^2^^ = 99.36 %, *P* < 0.0001; DL: *I*^^2^^ = 86.94 %, *P* = 0.0005; Fig. 4). Additional diagnostic parameters, including accuracy, specificity, sensitivity, PPV, and NPV, are detailed in Table S5.

## Performance of ML-based models in diagnosing different stages of MASH-related fibrosis

**Fig. 5:**
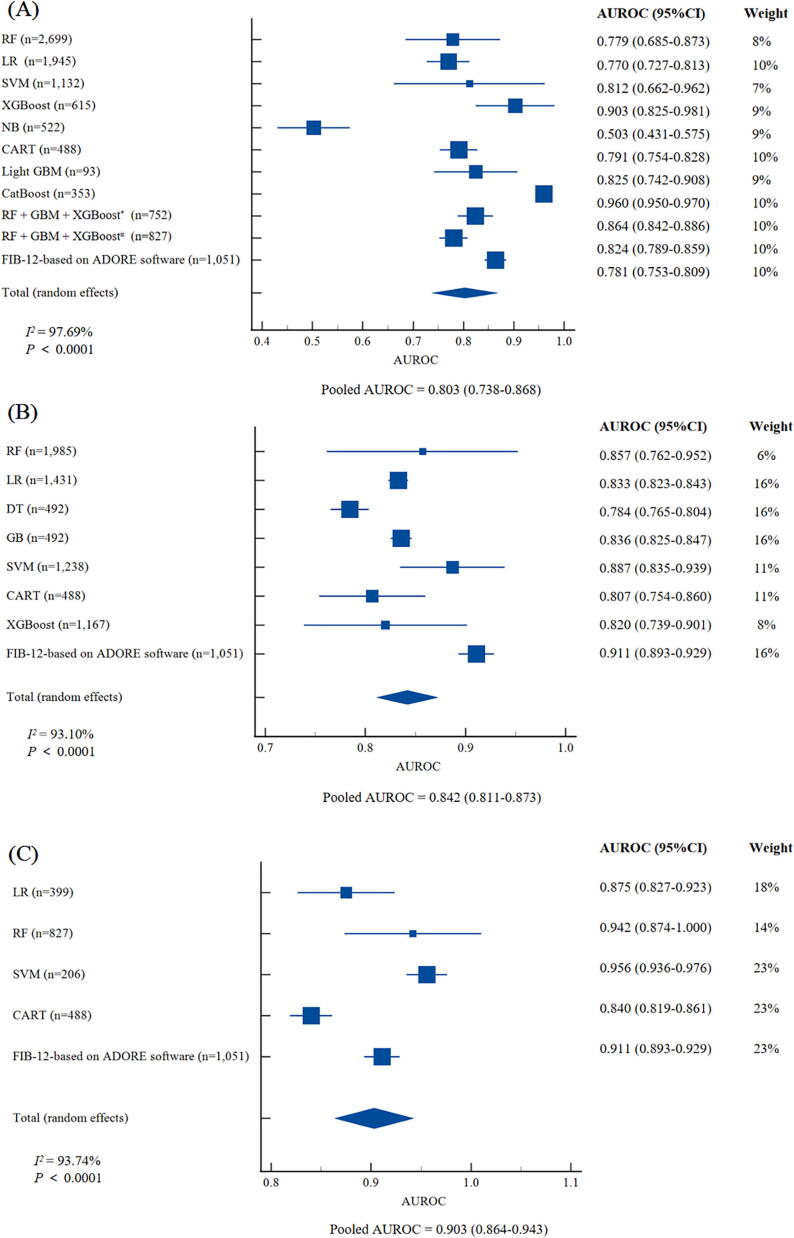
**Forest plots of AUROCs for diagnosing different liver fibrosis stages based on machine learning models. (A) Significant fibrosis (≥F2); (B) Advanced fibrosis (≥F3); (C) Cirrhosis (F4).** Abbreviations: AUROC, area under the receiver operator characteristic curve; CI: confidence interval; CART, Classification And Regression Tree; DT, Decision Tree; GB Gradient Boosting; GBM, Gradient Boosting Machine; GP, Gaussian process; LR, Logistic Regression; NB, Naive Bayes; RF, Random forests; SVM, Support Vector Machines; XGBoost, eXtreme Gradient Boosting.

Specifically, subgroup analyses were conducted to evaluate ML models in diagnosing different stages of liver fibrosis among the MASH population. A total of 12 studies (15,299 individuals), 11 studies (10,581 individuals), and 6 studies (7,496 individuals) were included to evaluate the diagnostic performance of ML in significant fibrosis (≥F2), advanced fibrosis (≥F3), and cirrhosis (F4), respectively. For significant fibrosis, CatBoost still outperformed other ML models, achieving the highest AUROC at 0.960 (95 %CI: 0.950–0.970), and XGBoost was the next best-performing model with an AUROC of 0.903 (95 %CI: 0.825–0.981) (Fig. 5A). As for advanced fibrosis, Fibrosis-12 index (FIB-12) based on ADORE demonstrated the best performance with an AUROC of 0.911 (95 %CI: 0.893–0.929), and SVM obtained the second-highest AUROC of 0.887 (95 %CI: 0.835–0.939) (Fig. 5B). Regarding cirrhosis, SVM was recognized as the best ML model, achieving an AUROC of 0.956 (95 %CI: 0.936–0.976), trailed by RF, which had an AUROC of 0.942 (95 %CI: 0.874–1.000) (Fig. 5C).

## Subgroup analyses for ML-based models

### Performance of ML-based models by geographic regions

To account for potential demographic and ethnic differences across populations, subgroup analyses were conducted to compare the performance of ML models in studies conducted in Asian versus those in non-Asia regions. For diagnosing MASH, LightGBM showed the best AUROC of 0.920 (95 %CI: 0.916–0.924) in studies from Asian (Fig. S5A), whereas XGBoost obtained the highest AUROC of 0.923 (95 %CI: 0.889–0.957) in non-Asia studies (Fig. S5B). In diagnosing liver fibrosis, XGBoost and SVM both showed the highest AUROC of 0.853 in Asia studies without significant heterogeneity (*I^2^* = 40.48 %, *P* = 0.109) (Fig. S6A). In non-Asia studies, CatBoost demonstrated the best performance, with an AUROC of 0.960 (95 %CI: 0.950–0.970). However, the great heterogeneity still existed in these studies, with *I^2^* of 99.67 % (*P* ＜0.0001) (Fig. S6B).

### Performance of ML-based models by ensemble and non-ensemble methods

Given the rapid evolution of ML in recent decades, subgroup analyses were performed classified by the ensemble and non-ensemble methods. For ensemble methods, LightGBM, CatBoost, FIB-12 based on ADORE software, and RF obtained the highest AUROCs in diagnosing MASH, significant fibrosis, advanced fibrosis, and cirrhosis, respectively. Among non-ensemble methods, DT had the highest AUROC in diagnosing MASH, whereas SVM consistently maintained superiority over other ML models across all fibrosis stages (≥F2, ≥F3, and F4) (Fig. S7). Notably, heterogeneity was observed in most comparisons, except for the analysis of ensemble methods in cirrhosis diagnosis (*I^2^* = 0.00 %, Fig. S7G).

### Performance of ML-based models by the year of publication

As the temporal trend revealed the evolving change of ML models, subgroup analysis was also conducted based on publication year (pre-2022 vs. post-2022). For diagnosing MASH, GB yielded the highest AUROC (0.817, 95 %CI: 0.808–0.825) among studies published during 2013–2022, while LightGBM outperformed other models among 2023–2025 publications (Fig. S8A-B). SVM maintained the highest diagnostic AUROCs across all fibrosis stages (≥F2, ≥F3, and F4) in pre-2022 studies, while more recent publications (2023–2025) showed CatBoost, FIB-12 based on ADORE software, and LR as optimal for diagnosing significant fibrosis, advanced fibrosis, and cirrhosis, respectively (Fig. S8C–H). Only studies published between 2023 and 2025 for diagnosing advanced fibrosis and cirrhosis did not show significant heterogeneity (*I^2^* = 25.97 % for advanced fibrosis, Fig. S8F; *I^2^* = 0.00 % for cirrhosis, Fig. S8H).

## Discussion

This study enrolled 106 eligible studies for systematic review, of which 35 provided sufficient data for meta-analysis to compare the diagnostic performance of various ML and DL-based models in diagnosing MASH and its associated fibrosis. Overall, both ML and DL models achieved strong diagnostic performance, with pooled AUROCs exceeding 0.80. Among ML algorithms, LightGBM was recognized as the best-performing model for diagnosing MASH, while ResNet50, a subtype of CNN, performed best among DL models. Both of them approached AUROCs of nearly 0.9. For fibrosis diagnosis, CatBoost achieved the highest AUROC overall and remained the best for identifying significant fibrosis. The FIB-12 model, developed using the ADORE platform (a hybrid ML algorithm), demonstrated the best performance in diagnosing advanced fibrosis, while SVM showed its superior performance in detecting cirrhosis. In DL-based models for diagnosing fibrosis, CNN yielded the highest AUROC. These findings provide evidence for model selection and development in the future and underscore the potential of AI-driven models to enhance diagnostic accuracy in MASH and related fibrosis. ML and DL models show their strong potential to aid clinical decision-making and improve comprehensive management for patients with MASLD.

MASLD represents a progressive spectrum ranging from simple hepatic steatosis to MASH, fibrosis, and eventually cirrhosis or HCC. Both MASH and fibrosis have been recognized as independent risk factors for liver-related mortality [27,28]. Therefore, early and accurate identification of disease progression is crucial. Although liver biopsy remains the gold standard, its invasive nature limits its routine application. Previous studies have explored numerous scoring systems, such as FIB-4 and NFS, to risk-stratify patients with MASLD. However, their diagnostic accuracy remains suboptimal [29].

With the development of AI, ML and DL have demonstrated superiority in medical diagnostics. ML excels at uncovering hidden patterns and identifying complex relationships within large datasets. Our study found that LR was the most commonly used ML model. Unlike traditional regression methods, such as simple linear regression models, which are valued for their clear interpretability but show limited capability for complex data processing, optimized LR-based algorithms can handle complex, multicollinear, and high-dimensional data and often operate as black-box systems. DL models, on the other hand, offer powerful capabilities in handling images, multi-omics, and other unstructured data. They can automatically extract relevant features and construct models for disease classification and lesion detection [30].

AI-assisted models have shown their advantage over conventional scoring systems [[31], [32], [33]]. For example, D. Chang et al. [34] developed an RF model using 17 demographic and clinical features to diagnose MASH, achieving higher AUROC compared with traditional tools such as FibroScan, FIB-4, FibroScan-aspartate aminotransferase (FAST), and NFS. Moreover, recent studies have demonstrated that AI-assisted models achieve diagnostic accuracy comparable to experienced pathologists when interpreting histological features of MASH and fibrosis [35,36]. Our study further confirmed that ML- and DL-based models offer robust diagnostic performance for both MASH and fibrosis, with pooled AUROCs consistently exceeding 0.80.

In our meta-analysis, models constructed using LightGBM and XGBoost demonstrated the best performance in diagnosing MASH, while CatBoost showed its highest AUROC in diagnosing fibrosis. Interestingly, all these three algorithms are based on the Gradient Boosting framework using DT.

LightGBM is optimized for memory efficiency and computational speed. It reduces data dimensionality and accelerates training, making it well-suited for large-scale datasets. However, its performance may be overestimated in smaller datasets. In our analysis, only one study using LightGBM reported diagnostic metrics based on an internal validation cohort of 259 patients with 75 clinical features per subject, achieving an AUROC of 0.92 [29]. Given the limited sample size and lack of external validation, this result should be interpreted with caution.

XGBoost, another cutting-edge ML algorithm, is featured by regularization and tree pruning to reduce model complexity and prevent overfitting. It also supports parallel processing, which speeds up data processing. In our study, four cohorts with 2,119 individuals developed XGBoost-based models to diagnose MASH, achieving a pooled AUROC of 0.892 [29,[37], [38], [39]]. In addition, XGBoost also showed sound performance in diagnosing significant fibrosis and advanced fibrosis with AUROCs of 0.903 and 0.820, respectively, further supporting its strong diagnostic performance.

Differentiated from XGBoost and LightGBM, CatBoost is optimized for classification, regression, and ranking tasks and can natively handle various types of data, including numeric, categorical, and text data without manual preprocessing. It is particularly effective in managing missing values and reducing overfitting to improve model accuracy and efficiency. Stefanakis K et al. [40] developed a CatBoost-based model to identify MASH patients with different stages of fibrosis, which demonstrated high AUROCs in both the training and internal validation sets, outperforming traditional non-invasive tests such as FIB-4 and NFS.

In DL models, NN, CNN, and ANN were the most frequently employed, which demonstrates strong capabilities in processing imaging, histopathology, multi-omics, and other complex data. Our meta-analysis found that DL models consistently achieved high diagnostic performance, with AUROCs exceeding 0.80 and even reaching above 0.90, for the diagnosis of MASH and related fibrosis.

To further improve diagnostic performance and promote clinical applicability, researchers have explored integrated approaches by combining different ML or DL models or hybridizing ML and DL models into unified platforms or developing patented software [35,41,42]. These developments aim to support point-of-care diagnostics and facilitate real-time clinical decision-making. AI-based algorithms with high performance underscore their potential to accurately detect MASH and fibrosis, thus reducing reliance on invasive procedures and avoiding misdiagnosis.

Of note, feature selection plays a crucial role in model development. Our study showed that age, gender, and body mass index remain the most commonly used demographic variables in diagnostic modeling. Frequently incorporated serological biomarkers reflecting liver function include liver enzymes, albumin, platelet count, alkaline phosphatase, and total bilirubin. In addition, biomarkers within lipid and glucose profiles, such as low- and high-density lipoproteins and glycated hemoglobin, are widely used.

Given the substantial heterogeneity within the MASLD population, the integration of multi-omics data, including metabolomics, lipidomics, urinary steroid metabolome, fecal metaproteomics, and radiomics, offers a promising strategy for identifying novel predictors and elucidating disease mechanisms. For instance, Masarone M et al. [43] integrated 10 different ML or DL models to analyze serum metabolomics profiles and developed three ensemble models capable of distinguishing different fibrosis stages among patients with MASLD, achieving diagnostic accuracy ranging from 73.9 % to 96.8 %. By leveraging ML and DL methods, researchers can integrate complex, high-dimensional datasets to uncover key metabolic pathways and enhance the predictive performance of diagnostic models in MASLD.

Our study represents one of the first meta-analyses to systematically and comprehensively evaluate the application of ML and DL in diagnosing MASH and its related fibrosis through comprehensive literature review and data synthesis. It not only assessed the overall diagnostic performance of ML and DL models but also examined the performance of ML models across different fibrosis stages, geographic regions, ensemble and non-ensemble methods, and studies publication of the year.

However, several limitations should be acknowledged. First, substantial heterogeneity was observed in this meta-analysis. This may be attributed to differences in AI models used, population characteristics, such as demographic and clinical profiles, data collection protocols, and the limited number of eligible studies available for certain comparisons. Although we conducted subgroup analyses by geographic regions, method types (ensemble vs. non-ensemble), and publication year (pre-2022 vs. post-2022), significant heterogeneity persisted. Notably, similar findings have been reported in meta-analyses of AI models for other medical conditions, such as chronic obstructive pulmonary disease, brain metastasis, ventricular arrhythmias, and oral cancer [[44], [45], [46], [47]], suggesting a broader methodological challenge requiring further investigation. Furthermore, due to the lack of detailed diagnostic outcome data, including true positives, false positives, false negatives, and true negatives, we were unable to perform meta-regression to explore potential sources of heterogeneity or to conduct pooled analyses for sensitivity, specificity, and diagnostic odds ratios. This limits the depth of comparative evaluation. Third, most included studies lacked external validation, raising concerns about potential overfitting. Consequently, the reported diagnostic performance may be overestimated, limiting the generalizability of these models to broader clinical settings. Finally, few studies contained prognostic models with insufficient data that cannot be used for additional calculations and comparative analysis. Future studies with larger, diverse cohorts and independent external validation are needed to confirm the robustness and clinical utility of AI-based diagnostic models for MASH and fibrosis.

## Conclusions

In conclusion, this systematic review and *meta*-analysis confirmed that both ML and DL models demonstrate strong diagnostic performance in identifying MASH and MASH-related liver fibrosis, with DL models showing superiority compared to ML, especially in fibrosis diagnosis. Within the ML framework, LightGBM showed the best performance for MASH diagnosis, while CatBoost performed best for fibrosis detection. Among DL algorithms, CNN achieved the highest accuracy. Notably, ML models also demonstrated consistent performance across different fibrosis stages, highlighting their robustness and adaptability. As advanced, non-invasive diagnostic tools, ML and DL-based models have emerged as valuable assets in the prevention, early diagnosis, and risk stratification of MASLD. Their integration into clinical practice holds great promise for improving decision-making and advancing precision MASLD management.

## Compliance with ethics requirements

Ethics approval was not required for this research.

## Declaration of competing interest

The authors declare that they have no known competing financial interests or personal relationships that could have appeared to influence the work reported in this paper.
