# Supplementary material for: Application of machine learning and deep learning in metabolic dysfunction-associated steatotic liver disease: a systematic review and meta-analysis
Source: J Adv Res. 2025 Aug 22;83:933–46. doi: 10.1016/j.jare.2025.08.042 (PMC13131525; doi:10.1016/j.jare.2025.08.042)
Supplement: Supplementary Data 1 [file mmc1.docx]

**Supplementary material:**

**Application of Machine learning and deep learning in Metabolic dysfunction-associated steatotic liver disease: a systematic review and meta-analysis**

Huan Zhang^#1,2^, Xiangyu Wu^#1,2^, Wenjing Ni^#1,2^, Jiali Wu^1^, Sisi Zhou^3^, Leyao Jia^3^, Mingyang Jin^4^, Sitian Zhao^4^, Zhenyao Jiang^4^, Chao Wu^5,2^, Yuxiang Sun^6^, Junping Shi^*4^, Jie Li^*5,1,2^

1. Department of Infectious Diseases, Nanjing Drum Tower Hospital Clinical College of Nanjing University of Chinese Medicine, Nanjing, Jiangsu, China.

2. Institute of Viruses and Infectious Diseases, Nanjing University, Nanjing, Jiangsu, China.

3. Department of Infectious Diseases, Nanjing Drum Tower Hospital Clinical College of Nanjing Medical University, Nanjing, Jiangsu, China.

4. Department of Infectious Diseases, The Affiliated Hospital of Hangzhou Normal University, Hangzhou, Zhejiang, China.

5. Department of Infectious Disease, Nanjing Drum Tower Hospital, Affiliated Hospital of Medical School, Nanjing University, Nanjing, Jiangsu, China.

6. Jiangsu Key Laboratory of Integrated Traditional Chinese and Western Medicine for Prevention and Treatment of Senile Diseases, Institute of Translational Medicine, Medical College, Yangzhou University, Yangzhou, Jiangsu, China.

^#^ These authors contributed equally.

**Supplementary tables: 5**

**Supplementary figures: 8**

**Content**

[Search strategies for four database 4](#_Toc12523)

[(A) PubMed 4](#_Toc7464)

[(B) Embase 4](#_Toc985)

[(C) Cochrane Library 5](#_Toc1980)

[(D) Web Of Science 6](#_Toc26018)

[Table S1. Extracted content. 7](#_Toc6897)

[Table S2. Eligible 76 studies on application of machine learning models. 9](#_Toc10989)

[Table S3. Eligible 30 studies on application of deep learning models. 22](#_Toc21616)

[Table S4. Metrics of machine learning and/or deep learning-based models for diagnosing MASH. 27](#_Toc20468)

[Table S5. Metrics of machine learning and/or deep learning-based models for diagnosing liver fibrosis. 32](#_Toc10225)

[Figure S1. Distribution of studies applying machine learning and/or deep learning by the year of publication. 38](#_Toc26703)

[Figure S2. Quality assessment of 35 eligible studies by Quality Assessment of Diagnostic Accuracy Studies tool 2 (QUADAS-2). 39](#_Toc9719)

[Figure S3. Forest plots of AUROCs for diagnosing MASH in machine learning and deep learning models.....................................................................................................................40](#_Toc13169)

[Figure S4. Forest plots of AUROCs for diagnosing liver fibrosis in machine learning and deep learning models.............................................................................................................41](#_Toc31088)

[Figure S5. Forest plots of AUROCs for diagnosing MASH based on machine learning models across different geographic regions.. 42](#_Toc13151)

[Figure S6. Forest plots of AUROCs for diagnosing liver fibrosis based on machine learning models across different geographic regions. 43](#_Toc7230)

[Figure S7. Forest plots of AUROCs for diagnosing MASH and its related fibrosis based on machine learning models classified by ensemble and non-ensemble methods 44](#_Toc11851)

[Figure S8. Forest plots of AUROCs for diagnosing MASH and its related fibrosis based on machine learning models by the year of publications...........................................................45](#_Toc8213)

[References 46](#_Toc26905)

**Search strategies for four database**

1. **PubMed**

(("non alcoholic steatohepatitis"[tiab:~1] OR "nonalcoholic steatohepatitis"[tiab:~1] OR (NASH[tiab] AND liver) OR "metabolic dysfunction-associated steatohepatitis"[tiab:~1]OR "metabolic-associated steatohepatitis"[tiab:~1] OR (MASH[tiab]AND liver)) OR (("Non-alcoholic Fatty Liver Disease"[Mesh] OR ("Non-alcoholic Fatty Liver Disease"[Mesh:noexp] OR "non alcoholic fatty liver"[tiab:~1] OR "nonalcoholic fatty liver"[tiab:~1] OR NAFLD[tw] OR "non alcoholic steatohepatitis"[tiab:~1] OR "nonalcoholic steatohepatitis"[tiab:~1] OR (NASH[tiab] AND liver)) OR ("metabolic dysfunction-associated fatty liver disease"[tiab:~1] OR "metabolic dysfunction associated fatty liver disease"[tiab:~1] OR "metabolic-associated fatty liver disease"[tiab:~1] OR "metabolic associated fatty liver disease"[tiab:~1] OR MAFLD[tw] OR (MAFLD[tiab] AND liver)) OR ("metabolic dysfunction-associated steatotic liver disease"[tiab:~1] OR "metabolic dysfunction associated steatotic liver disease"[tiab:~1] OR MASLD[tw] OR "metabolic dysfunction-associated steatohepatitis"[tiab:~1]OR "metabolic-associated steatohepatitis"[tiab:~1] OR (MASH[tiab]AND liver))) AND ("fibrosis" [tiab] OR "cirrhosis" [tiab]))) AND (“Artificial Intelligence”[mesh] OR “Artificial Intelligence”[tiab:~1] OR “AI”[tiab] OR “pathAI”[tiab] OR “Histolndex”[tiab] OR “PharmaNest”[tiab] OR “Biocellvia”[tiab] OR “FibroNest”[tiab] OR “MorphoQuant”[tiab] OR “dual-photon microscopy”[tiab] OR “digital pathology”[tiab] OR “radiomics”[tiab] OR “Computational Intelligence”[tiab] OR “Machine Intelligence”[tiab] OR “Computer Reasoning”[tiab] OR “Computer Vision”[tiab] OR “Machine learning”[tiab] OR “Deep Learning”[tiab] OR “Supervised Machine learning”[tiab] OR “Unsupervised Machine learning”[tiab] OR “Support Vector Machine”[tiab] OR “Support Vector Machines”[tiab] OR “Support Vector Network”[tiab] OR “Support Vector Networks”[tiab] OR “SVN”[tiab] OR “Decision Trees”[tw] OR “Decision Tree”[tiab] OR “Random Forest”[tiab] OR “Random Forests”[tiab] OR “Cluster Analysis”[tiab] OR “Cluster Analyses”[tiab] OR “Clustering”[tiab] OR “Clusterings”[tiab] OR “Neural network”[tiab] OR “Convolutional Neural Network”[tiab] OR “CNN”[tiab] OR “Artificial Neural Network”[tiab] OR “ANN”[tiab] OR “Ensemble learning”[tiab] OR “Naive Bayes”[tiab] OR “K-Nearest Neighbor”[tiab] OR “KNN”[tiab] OR “K-means”[tiab] OR “Long Short-Term Memory Networks”[tiab] OR “Autoencoder”[tiab] OR “Generative Adversarial Network”[tiab] OR “XG-Boost”[tiab] OR “XGB”[tiab] OR “linear regression”[tiab] OR “Logistic Regression”[tiab] OR “Deep Convolutional Neural Network”[tiab] OR “DCNN”[tiab] OR “residual network”[tiab]) AND (“predict*”[tiab] OR “prognos*”[tiab] OR “diagnos*”[tiab] OR "incident"[tiab] OR “detect*”[tiab] OR “classif*”[tiab]) NOT (animals[mesh] NOT humans[mesh]) NOT ((infant[mesh] OR child[mesh] OR adolescent[mesh]) NOT adult[mesh]) NOT (comment[pt] OR letter[pt] OR Bibliography[pt] OR editorial[pt] OR news[pt] OR "rats"[ti])

1. **Embase**

((nash:ti,ab AND liver:ti,ab,kw) OR (mash:ti,ab AND liver:ti,ab,kw) OR (('nonalcoholic fatty liver'/exp OR ((('non alcoholic' OR 'nonalcoholic') NEAR/2 fatty NEAR/2 (liver OR hepat* OR steatohepatitis)):ti,ab) OR nafld:ti,ab,kw,de OR (nash:ti,ab AND liver:ti,ab,kw) OR 'metabolic dysfunction associated fatty liver'/exp OR ((('metabolic dysfunction associated' OR 'metabolic associated') NEAR/2 fatty NEAR/2 (liver OR hepat* OR steatohepatitis)):ti,ab) OR mafld:ti,ab,kw,de OR 'metabolic dysfunction associated steatotic liver'/exp OR ((('metabolic dysfunction associated') NEAR/2 steatotic NEAR/2 (liver OR hepat* OR steatohepatitis)):ti,ab) OR masld:ti,ab,kw,de OR (mash:ti,ab AND liver:ti,ab,kw)) AND (‘fibrosis’:ti,ab,kw OR ‘cirrhosis’:ti,ab,kw))) AND (‘Artificial Intelligence'/exp OR ‘Artificial Intelligence’:ti,ab,kw OR ‘AI’:ti,ab,kw OR ‘pathAI’:ti,ab,kw OR ‘Histolndex’:ti,ab,kw OR ‘PharmaNest’:ti,ab,kw OR ‘Biocellvia’:ti,ab,kw OR ‘FibroNest’:ti,ab,kw OR ‘MorphoQuant’:ti,ab,kw OR ‘dual-photon microscopy’:ti,ab,kw OR ‘digital pathology’:ti,ab,kw OR ‘radiomics’:ti,ab,kw OR ‘Computational Intelligence’:ti,ab,kw OR ‘Machine Intelligence’:ti,ab,kw OR ‘Computer Reasoning’:ti,ab,kw OR ‘Computer Vision’:ti,ab,kw OR ‘Machine learning’:ti,ab,kw OR ‘Deep Learning’:ti,ab,kw OR ‘Supervised Machine learning’:ti,ab,kw OR ‘Unsupervised Machine learning’:ti,ab,kw OR ‘Support Vector Machine’:ti,ab,kw OR ‘SVM’:ti,ab,kw OR ‘Support Vector Machines’:ti,ab,kw OR ‘Support Vector Network’:ti,ab,kw OR ‘Support Vector Networks’:ti,ab,kw OR ‘Decision Trees’:ti,ab,kw OR ‘Decision Tree’:ti,ab,kw OR ‘Random Forest’:ti,ab,kw OR ‘Random Forests’:ti,ab,kw OR ‘Cluster Analysis’:ti,ab,kw OR ‘Cluster Analyses’:ti,ab,kw OR ‘Clustering’:ti,ab,kw OR ‘Clusterings’:ti,ab,kw OR ‘Neural network’:ti,ab,kw OR ‘Convolutional Neural Network’:ti,ab,kw OR ‘CNN’:ti,ab,kw OR ‘Artificial Neural Network’:ti,ab,kw OR ‘ANN’:ti,ab,kw OR ‘Ensemble learning’:ti,ab,kw OR ‘Naive Bayes’:ti,ab,kw OR ‘K-Nearest Neighbor’:ti,ab,kw OR ‘KNN’:ti,ab,kw OR ‘K-means’:ti,ab,kw OR ‘Long Short-Term Memory Networks’:ti,ab,kw OR ‘Autoencoder’:ti,ab,kw OR ‘Generative Adversarial Network’:ti,ab,kw OR ‘GAN’:ti,ab,kw OR ‘XG-Boost’:ti,ab,kw OR ‘XGB’:ti,ab,kw OR ‘linear regression’:ti,ab,kw OR ‘Logistic Regression’:ti,ab,kw OR ‘Deep Convolutional Neural Network’:ti,ab,kw OR ‘DCNN’:ti,ab,kw OR ‘residual network’:ti,ab,kw) AND (‘predict*’:ti,ab,kw OR ‘prognos*’:ti,ab,kw OR ‘diagnos*’:ti,ab,kw OR ‘incident’:ti,ab,kw OR ‘detect*’:ti,ab,kw OR ‘classif*’:ti,ab,kw) NOT ('animal'/exp NOT 'human'/exp) NOT (('juvenile'/exp OR 'child'/exp OR 'adolescent'/exp) NOT 'adult'/exp) NOT 'review'/exp NOT 'rats':ti NOT ([conference abstract]/lim OR [conference review]/lim OR [editorial]/lim OR [letter]/lim OR [note]/lim)

1. **Cochrane Library**

((“NASH” AND (hepatic OR liver OR steatohepatitis)) OR ((“Metabolic dysfunction associated steatotic” OR “MASH”) AND (hepatic OR liver OR steatohepatitis)) OR ((((“Non alcoholic fatty” OR “nonalcoholic fatty” OR “NASH”) AND (hepatic OR liver OR steatohepatitis)) OR “NAFLD”) OR (((“Metabolic dysfunction associated” OR “metabolic associated”) AND (hepatic OR liver)) OR “MAFLD”) OR (((“Metabolic dysfunction associated steatotic” OR “MASH”) AND (hepatic OR liver OR steatohepatitis)) OR “MASLD”) AND ("fibrosis" OR "cirrhosis"))) AND (“Artificial Intelligence” OR “AI” OR “pathAI” OR “Histolndex” OR “PharmaNest” OR “Biocellvia” OR “FibroNest” OR “MorphoQuant” OR “dual-photon microscopy” OR “digital pathology” OR “radiomics” OR “Computational Intelligence” OR “Machine Intelligence” OR “Computer Reasoning” OR “Computer Vision” OR “Machine learning” OR “Deep Learning” OR “Supervised Machine learning” OR “Unsupervised Machine learning” OR “Support Vector Machine” OR “SVM” OR “Support Vector Machines” OR “Support Vector Network” OR “Support Vector Networks” OR “Decision Trees” OR “Decision Tree” OR “Random Forest” OR “Random Forests” OR “Cluster Analysis” OR “Cluster Analyses” OR “Clustering” OR “Clusterings” OR “Neural network” OR “Convolutional Neural Network” OR “CNN” OR “Artificial Neural Network” OR “ANN” OR “Ensemble learning” OR “Naive Bayes” OR “K-Nearest Neighbor” OR “KNN” OR “K-means” OR “Long Short-Term Memory Networks” OR “Autoencoder” OR “Generative Adversarial Network” OR “GAN” OR “XG-Boost” OR “XGB” OR “linear regression” OR “Logistic Regression” OR “Deep Convolutional Neural Network” OR “DCNN” OR “residual network”) AND (predict* OR prognos* OR diagnos* OR incident OR detect* OR classif*)

**(D) Web Of Science**

(TS=((nash AND liver) OR (mash AND liver) OR (((("non alcoholic" OR "nonalcoholic") NEAR/2 fatty NEAR/2 (liver OR hepat* OR steatohepatitis)) OR nafld OR (nash AND liver) OR (("metabolic dysfunction associated" OR "metabolic associated") NEAR/2 fatty NEAR/2 (liver OR hepat* OR steatohepatitis)) OR mafld OR (("metabolic dysfunction associated") NEAR/2 steatotic NEAR/2 (liver OR hepat* OR steatohepatitis)) OR masld OR (mash AND liver)) AND ("fibrosis" OR "cirrhosis"))) AND (TS=(“Artificial Intelligence” OR “AI” OR “pathAI” OR “Histolndex” OR “PharmaNest” OR “Biocellvia” OR “FibroNest” OR “MorphoQuant” OR “dual-photon microscopy” OR “digital pathology” OR “radiomics” OR “Computational Intelligence” OR “Machine Intelligence” OR “Computer Reasoning” OR “Computer Vision” OR “Machine learning” OR “Deep Learning” OR “Supervised Machine learning” OR “Unsupervised Machine learning” OR “Support Vector Machine” OR “SVM” OR “Support Vector Machines” OR “Support Vector Network” OR “Support Vector Networks” OR “Decision Trees” OR “Decision Tree” OR “Random Forest” OR “Random Forests” OR “Cluster Analysis” OR “Cluster Analyses” OR “Clustering” OR “Clusterings” OR “Neural network” OR “Convolutional Neural Network” OR “CNN” OR “Artificial Neural Network” OR “ANN” OR “Ensemble learning” OR “Naive Bayes” OR “K-Nearest Neighbor” OR “KNN” OR “K-means” OR “Long Short-Term Memory Networks” OR “Autoencoder” OR “Generative Adversarial Network” OR “GAN” OR “XG-Boost” OR “XGB” OR “linear regression” OR “Logistic Regression” OR “Deep Convolutional Neural Network” OR “DCNN” OR “residual network”)) AND (TS=(“predict*” OR “prognos*” OR “diagnos*” OR "incident" OR “detect*” OR “classif*”)) NOT TI=("rats") NOT (DT==("Meeting Abstract" OR "Editorial Material" OR "Letter" OR "Book Chapter"))

**Supplementary appendix**

**Table S1. Extracted content.**

| **Domain** | **Items** |
| --- | --- |
| SOURCE OF DATA | Source of data |
| PARTICIPANTS | Participant eligibility and recruitment method |
|  | Details of treatments received, if relevant |
|  | Study dates |
| OUTCOME(S) TO BE PREDICTED | Definition and method for measurement of outcome |
|  | Was the same outcome definition (and method for measurement) used in all patients? |
|  | Type of outcome |
|  | Was the outcome assessed without knowledge of the candidate predictors? |
|  | Were candidate predictors part of the outcome? |
| CANDIDATE PREDICTORS (OR INDEX TESTINGS) | Number and type of predictors |
|  | Definition and method for measurement of candidate predictors |
|  | Timing of predictor measurement |
|  | Were predictors assessed blinded for outcome, and for each other (if relevant)? |
|  | Handling of predictors in the modelling |
| SAMPLE SIZE | Number of participants and number of outcomes/events |
| MISSING DATA | Number of participants with any missing value (include predictors and outcomes) |
|  | Number of participants with missing data for each predictor |
|  | Handling of missing data (e.g., complete-case analysis, imputation, or other methods) |
| MODEL DEVELOPMENT | Modelling method |
|  | Method for selection of predictors for inclusion in multivariable modelling |
|  | Method for selection of predictors during multivariable modelling and criteria used |
|  | Shrinkage of predictor weights or regression coefficients |
| MODEL PERFORMANCE | Calibration (calibration plot, calibration slope, Hosmer-Lemeshow Testing) and Discrimination (C-statistic, D-statistic, log-rank) measures with confidence intervals |
|  | Classification measures and whether a-priori cut points were used |
| MODEL EVALUATION | Method used for Testing model performance: development dataset only (random split of data, resampling methods) or separate External validation validation |
|  | In case of poor validation, whether model was adjusted or updated |
| RESULTS | Final and other multivariable models presented, including predictor weights or regression coefficients, intercept, baseline survival, model performance measures (with standard errors or confidence intervals) |
|  | Any alternative presentation of the final prediction models |
|  | Comparison of the distribution of predictors (including missing data) for development and validation datasets |
| INTERPRETATION AND DISCUSSION | Interpretation of presented models |
|  | Comparison with other studies, discussion of generalizability, strengths and limitations. |

**T****able S2.** **Eligible 76 studies on application of machine learning models****.**

| **Study** | **Outcomes** | | **Study country/area** | | **Algorithms^△^** | **Participants**  **(No.)** | **Training size (No.)** | **Testing size (No.)** | **Validation size (No.)** | **Used features/**  **Supplementary information** |  |
| --- | --- | --- | --- | --- | --- | --- | --- | --- | --- | --- | --- |
| Anushiravani et al 2023[1] | Fibrosis | | Iran, Kingdom of Saudi Arabia (KSA), Egypt, Greece, Turkey, and Oman | | RF | 2,472 | NR | NR | 2,472 | Age, ALT, AST, ALP, albumin, and PLT |  |
| Ben-Assuli et al 2022[2] | Fibrosis | | NR | | Time-series clustering ML models | 5,579 | NR | NR | NR | Age, gender, waist, BMI, smoking status, AST, ALT, GGT, TC, HDL -C, TG, HbA1c, high sensitive CRP, PLT, UA, albumin, bilirubin, ALP, hypertension, diabetes, metabolic syndrome, hypertriglyceridemia, HDL-C< LCL |  |
| *Canbay et al 2019[3] | Fibrosis | | NR | | LR | 286 | 164 | NR | 122(Internal validation) | Age, GGT, HbA1c, adiponectin, and M30 |  |
| *Eslam et al 2016[4] | Fibrosis | | Multiple countries | | Decision Tree | 4,277 | 1,992 | NR | 2,285(External validation) | Interferon-k4 gene-IFNL genotype, age, gender and routinely assessed clinical and laboratory variables |  |
| Fialoke et al 2018[5] | Fibrosis | | NR | | XGBoost | NR | NR | NR | NR | Age, hyperglycemia, BMI, PLT, albumin and the AST/ALT ratio |  |
| Ghadiri et al 2022[6] | Fibrosis | | USA | | KNN | 365 | 365 | NR | NR | Two single nucleotide polymorphisms in the cytochrome P450 family 2 subfamily E member 1 gene (rs6413432, rs3813867), two SNPs in the glucokinase regulator gene (rs780094, rs1260326), rs738409 single nucleotide polymorphism in patatinlike phospholipase domain-containing 3, and gender parameters |  |
| Lee et al 2023[7] | Fibrosis | | Netherlands | | GBM | 966 | 966 | NR | NR | Gender, age, BMI, WC, systolic blood pressure, diastolic blood pressure, T2DM, HDL, LDL, TG, cholesterol, ALT, AST, GGT, ALP, PLT, hemoglobin, transferrin saturation, albumin, ferritin, clotting (prothrombin time), bilirubin, glucose, fasting insulin, HbA1c, fibrinogen |  |
| Loomba et al 2017[8] | Advanced fibrosis | | | USA | RF and SVM | 86 | 86 | NR | NR | Fecal-microbiome derived metagenomic signature |  |
| Schattenberg et al2023[9] | Fibrosis | | | NR | XGBoost | NR | NR | NR | NR | HbA1c, AST, ALT, total protein, AST/ALT, BMI, TG, height,  PLT, WBC, hematocrit, albumin, hypertension, gender |  |
| Sokolowska et al 2022[10] | Fibrosis | | | Poland | Tree-based automated ML | 18 | 18 | NR | NR | Dietary adherence, BMI, weight, blood and stool biochemistry. |  |
| *Verma et al 2024[11] | Significant fibrosis | | 8 Asia countries | | RF | 1,656 | 1,656 | NR | NR | Age, aminotransferase levels, PLT, fasting plasma glucose, diabetes control, and GGT. |  |
| *Sowa et al 2013[12] | Fibrosis | | Germany | | RF | 126 | 126 | NR | NR | ALT, AST, M30, M60, HA |  |
| Charu et al 2023[13] | Fibrosis | | US | | SuperLearner | 2,162 | 648 | NR | 1514 (External validation) | Age, sex, race, Hispanic ethnicity, T2DM, hypertension, albumin, ALT, ALP, AST, BMI, GGT, globulin, fasting glucose, HDL-c, hematocrit, HbA1c, LDL-c, PLT, total bilirubin, TC, TG, and WBC |  |
| Hassoun et al 2024[14] | Fibrosis | | Italy | | SVM | 5,784 | 5,381 | | 283 (Internal validation)  120 (External validation) | GGT, glycohemoglobin, C-reactive protein glucose, UA, HDL-C, ALT, triglycerides, age, AST, ferritin, blood albumin, ALP, percentage of lymphocytes, total count of white cells, percentage of segmented neutrophils, platelet count, total bilirubin, sex, hematocrit, hemoglobin |  |
| Brausch et al 2021[15] | Fibrosis | | Germany | | SVM | 27 | 27 | NR | NR | / |  |
| *Chen et al 2021[16] | Fibrosis | | NR | | LR | 22 | 22 | NR | NR | Radiomics features of 18F-FDG PET images |  |
| *Fan et al 2024[17] | Fibrosis | | China, USA | | LR | 946 | 946 | NR | NR | Liver stiffness measurement, age, sex, PLT, albumin, total bilirubin |  |
| *Feng et al 2021[18] | Fibrosis | | China | | RF | 553 | 553 | NR | 275 (Internal validation) | BMI, pro-collagen type III, collagen type IV, AST, A/G ratio |  |
| Goldman et al 2021[19] | Fibrosis | | Israel | | CHAID Decision Tree | 12,019 | 12,019 | NR | NR | Demographics, athletics habits & exercise Testing results, metabolic components (number of components of the metabolic syndrome, waist-hip ratio, BMI, glucose and HbA1c blood Testings), routine blood counts, chemistry and lipid profile, history of hypertension, dyslipidemia, cardiovascular diseases, medication intake |  |
| Meng et al 2023[20] | Fibrosis | | China | | SVM | 618 | 432 | 186 | NR | Three radiomics features from shear wave elastography and two from grayscale imaging |  |
| *Moolla et al 2020[21] | Fibrosis | | NR | | GMLVQ | 275 | 275 | NR | NR | Urinary steroid metabolome |  |
| Sripongpun et al 2022[22] | Fibrosis | | USA | | LR | 676 | 676 | NR | NR | Age, BMI, diabetes, AST, ALT, globulin, PLT |  |
| Suárez et al 2023[23] | Fibrosis | | Mexico | | XGBoost | 407 | 407 | NR | NR | Platelet level, dyslipidaemia, T2DM |  |
| Suárez et al 2023[24] | Fibrosis | | Mexico | | Adaboost | 211 | 211 | NR | NR | Platelet level, dyslipidaemia, T2DM |  |
| Suárez et al 2023[25] | Fibrosis | | Mexico | | XGBoost | 215 | 215 | NR | NR | HDL-c, systemic arterial hypertension and TG |  |
| *Wang et al 2019[26] | Fibrosis | | China | | ML# | 344 | 206 | 138 | NR | / |  |
| *Bastati et al 2023[27] | MASH | | NR | | RF-based on UDC | 76 | 46 | NR | 30 (Internal validation) | TBil, ALT, AST, ALP, GGT, TG, HDL-C, glucose |  |
| Naderi Yaghouti et al 2024[28] | MASH | | Japan | | RF | 176 | 176 | NR | NR | GPT, GGGT, TG, BMI, HbA1c, Lp(a), Loge (Lp(a)), HDL, LDL, Ezetimibe |  |
| Sydor et al 2022[29] | MASH | | Germany | | ML# | 80 | 80 | NR | NR | Fecal metaproteomics |  |
| Yasar et al 2022[30] | MASH | | NR | | Gradient boosted trees using the XGBoost package | 1,463,089 | 1,463,089 | NR | NR | Age, sex, type 2 diabetes, knowledge-driven features, data-driven features |  |
| Yilmaz et al 2012[31] | MASH | | Turkey | | SVM | 136 | 136 | NR | NR | Osteoprotegerin, fibroblast growth factor 21, and M30. |  |
| Zhu et al 2022[32] | MASH | | China | | RF | 83 | 83 | NR | NR | 4 lipid molecules: free fatty acid (18:0), lysophosphatidylcholine (22:6/0:0), free fatty acid (18:1), and phosphatidylinositol (16:0/18:1) |  |
| Jiang et al 2023[33] | MASH | | NR | | RF | 91 | 48 | NR | 43 (Internal validation) | Amidolytic domain1 1, FMO1, Lipoprotein lipase, and Proline 4-hydroxylase subunit α1. |  |
| Ji et al 2022[34] | MASH | | South Korea | | RF and LR | 86 | 86 | NR | NR | Glutamic acid, isocitric acid, and aspartic acid |  |
| Carr et al 2023[35] | | Non-cirrhotic MASH | USA | | RF | 279 | 279 | NR | NR | Fecal bile acids |  |
| Barbois et al 2021[36] | | MASH | France | | Decision Tree | 360 | 102 | NR | 258 | ALT |  |
| Docherty et al 2021[37] | | MASH | USA | | XGBoost | ~3,000,000 | 422 | 282 | ~3,000,000 | HbA1c, AST, ALT, total protein AST/ALT, BMI, TG, height, platelets, WBC, hematocrit albumin, hypertension, gender |  |
| García-Carretero et al 2021[38] | | MASH | Spain | | RF | 1525 | 1525 | NR | NR | Insulin resistance, ferritin, serum levels of insulin, and TG |  |
| Luo et al 2021[39] | | MASH | USA | | Elastic-Net algorithm | 216 | 113 | NR | 103 | Latent transforming growth factor beta binding protein 4, insulin-like growth factor 1, vascular cell adhesion molecule 1, interleukin-1 soluble receptor type-1, interleukin-18-binding protein, thrombospondin-2, collectin kidney 1, sex hormone–binding globulin;, interleukin-27 receptor subunit alpha, leukemia inhibitory factor receptor, soluble, fibulin-3, and plexin-B2 |  |
| Masarone et al 2021[40] | MASH | | Italy | | ML# | 307 | 307 | NR | NR | Bile acids, lipid and amino acid metabolism |  |
| [Noureddin](https://pubmed.ncbi.nlm.nih.gov/?term=Noureddin+M&cauthor_id=36647687) et al 2023[41] | MASH | | NR | | MATLAB | 143 | 143 | NR | NR | 448 histologic variables (243 related to septa, 21 related to nodules and 184 related to fibrosis) |  |
| Teramoto et al 2020[42] | MASH | | Japan | | LR | 79 | 79 | NR | NR | / |  |
| *Chang et al 2021[43] | Fibrosis/ MASH with significant fibrosis | | USA | | RF | 1,370 | 1,370 | NR | NR | Gender, age, BMI, ALP, TBil, ALT, AST, albumin, WBC, PLT, HbA1c, TC, LDL, HDL, TG, T2DM, and hypertension status |  |
| Perakakis et al 2019[44] | | MASH/  Fibrosis | Greece | | SVM | 80 | 80 | NR | NR | Lipids, hormones and glycans |  |
| *Wu et al 2022[45] | | MASH/  Fibrosis | USA | | GB | 492 | 492 | NR | NR | AST, ALT and TG are important risk factors for NASH.  AST, HbA1c and HDL are important variables in predicting advanced fibrosis. |  |
| Mamandipoor et al 2023[46] | MASH | | Austria | | XGBoost | 5,834 | 5,834 | NR | NR | Age, gender, weight, height, BMI, ALT, AST, AP, Bilirubin, Cholinestherase, GGT, Blood_glucose, Cholesterol, C-reactive protein, Uric acid, HDL cholesterol, hemoglobin, LDH, LDL cholesterol, Platetelets, Triglycerides, TSH, INR, Lipase, Amylase, Baso, Blood sedation rate, Iron, Ferritin, Transferrin saturation, Transferrin, Albumin, HOMA-IR, Oral glucose tolerance Testing, alcohol per day, coffee cups, vegetable portions, sugar sweetened beverages, red meat consumption, fast food meals, fruits porition, current or past smoker, systolic arterial pressure, diastolic arterial pressure, ACE Inhibitors, Statins, Family history, aspirin use |  |
| *Njei et al 2024[47] | MASH | | USA | | XGBoost | 5,156 | 1,719 | 1,718 | 1,719 (Internal validation) | ALT, GGT, Platelet count, Waist circumference, Age |  |
| Sarkar et al 2024[48] | Hepatocellular carcinoma | | NR | | Gradient boosted | 2,247 | 1,561 | NR | 686 (External validation) | Age, sex, race, ethnicity, diabetes, hypertension, obesity, , creatinine, AKP, bilirubin, albumin, cholesterol, FIB-4 score, prothrombin time/INR, sodium, potassium, chloride |  |
| Baser et al 2024[49] | MASH | | USA | | RF | 4,223,443 | 4,223,443 | NR | NR | Age, obesity, abnormal liver function Testings et al |  |
| Charu et al 2024[50] | Fibrosis | | NR | | Superlearner | 2,162 | 648 | NR | 1,514 (External validation) | Age, sex, race (White vs. other), Hispanic ethnicity, type II diabetes status, hypertension, albumin, ALT AKP, AST, BMI, GGT, globulin, fasting glucose, HDL cholesterol, hematocrit, hemoglobin A1C, LDL cholesterol, platelets, total bilirubin, total cholesterol, triglycerides, and white blood cell count |  |
| Chen et al 2024[51] | MASH | | China | | Boruta analysis | 250 | 250 | NR | NR | Total protein, AST, ALT, insulin, 10 metabolites (e.g., heptadecanoic acid, citrate),  3 lipid species (specific TG and PC species). |  |
| *Dabbah et al 2025[52] | Fibrosis | | Israel | | XGBoost | 1,158 | 618 | NR | 540 (Internal validation) | Age, sex, BMI, DM/impaired fasting  glucose, HbA1c, hypertension, AST, ALT, GGT, ALP, platelets, albumin, total cholesterol, LDL-c, HDL-c, TG |  |
| Davidov et al 2024[53] | MASH/  Fibrosis | | Israel | | Bagged trees | 46 | 46 | NR | NR | 38 thermal imaging features |  |
| *Feng et al 2024[54] | Fibrosis | | China | | LR | 571 | 399 | NR | 172 (External validation) | αC18:3, γ-C18:3, sex, age, BMI, SBP, duration of diabetes, Triglyceride glucose index, HDL-C, HbA1c, fasting c-peptide, and c-peptide 2 h postprandial |  |
| Lu et al 2024[55] | Fibrosis | | China | | XGBoost | 194 | 135 | NR | 59 (Internal validation) | DM status, AST, ALT, Ultrasonographic fibrosis score, LSM |  |
| *Mouskeftara et al 2024[56] | MASH | | Greece | | XGBoost | 37 | 37 | NR | NR | HOMA-IR, BMI, platelet count, LDL-c, ferritin, AST, FA 12:0, FA 18:3 伪3, FA 20:4/FA 20:5 ratio, CAR 4:0, LPC 20:4, LPC O-16:1, LPE 18:0, DG 18:1_18:2, CE 20:4 |  |
| Blomdahl et al 2025[57] | F0-1 vs, F2-4 | | Sweden | | RF | 119 | 60 | NR | 35 (Internal validation)/24 (External validation) | IGFBP-7, angiotensin  converting enzyme-2, hepatocyte growth factor |  |
| *Snethlage et al 2024[58] | Fibrosis | | Netherlands | | Extra-Trees classification | 453 | 453 | NR | NR | type 1 diabetes duration, age, sex, BMI, stimulated  C-peptide/creatinine ratio, SBP, daily insulin dose, time below range/time in range/time above range of glucose, and HbA1c |  |
| *Zamanian et al 2024[59] | MASH | | Japan | | RF | 176 | 176 | NR | NR | BMI, ALT, TC,HDL, Ezetimibe, lipoprotein level Lp(a), Loge(Lp(a)), TG, Creatinine, HbA1c, Fibrate, and Sex |  |
| *Yan et al 2024[60] | MASH | | China | | XGBoost | 587 | 406 | NR | 181 (External validation) | Neutrophil percentage, AST/ALT ratio, hematocrit, creatinine, uric acid, prealbumin |  |
| Chen et al 2025[61] | MASH | | China | | RF | 558 | 390 | NR | 168 (Internal validation) | BMI, weight, height, ALT, AST, PLT, uric acid, creatinine, fasting insulin, DM, HbA1c, hypertension, HDL-C, LDL-C, TG, ALB, total bilirubin, TC, sex, age. |  |
| *Huang et al 2025[62] | MASH/  Mortality | | China , Finland | | RF | 118,182 | 117,877 | NR | 305 (External validation) | BMI, AST, Tyrosine, phospholipid-to-total lipid ratio in VLDL |  |
| *Liu et al 2025[63] | MASH/  Fibrosis | | China | | XGBoost | 561 | 456 | NR | 105 (External validation) | AST: ALT ratio, homeostatic model assessment, fibrotic non-alcoholic steatohepatitis index, BMI, diabetes, white blood cell, neutrophil, basophil, PLT, APTT, fibrinogen, insulin, ALT, AST, GGT, HbA1c |  |
| Matholi et al 2025[64] | MASH | | Egypt | | RF | 297 | 237 | 60 | N/R | EP300 mRNA, CPN60 mRNA, AST, D. bilirubin, Albumin, GGT, HbA1c, HOMA-IR, and BMI | |
| Mounika et al 2025[65] | MASH | | India | | RF | 233 | 233 | NR | NR | G-CSF, IL-9, IL-13, eotaxin, and TNF-α |  |
| *Alkhouri et al 2025[66] | Fibrosis | | USA, Hong Kong, France, Australia, India, etc. | | RF, gradient boosting machines, and XGBoost | 3,630 | 827 | 1,504 | 1,299 (External validation) | Age, sex, BMI, lab parameters (AST, ALT, platelet, GGT, etc.), VCTE. |  |
| Boullion et al 2025[67] | Fibrosis | | USA | | RF | 3857 | 3,086 | 771 | NR | Age, sex, race, BMI, HbA1C, plasma fasting glucose, insulin, TC, LDL, HDL, triglycerides, ALT, AST, ALP, albumin, GGT, lactate dehydrogenase, iron, bilirubin, total protein, uric acid, blood urea nitrogen, hs-CRP. |  |
| *Panagiotopoulos et al 2025[68] | MASH/  Fibrosis | | USA | | Bayesian Information Criterion-based stepwise logistic regression | 140 | 140 | NR | NR | PDFF, AST, and sex (MASH);  PDFF and MRE (Fibrosis) |  |
| *Stefanakis et al 2025[69] | MASH and F2-F3 | | USA, Italy, Australia, Greece | | Categorical Gradient Boosting Machines | 443 | 353 | NR | 90 (Internal validation) | ALT, AST, BMI, metabolic syndrome components, 3-ureidopropionate, alpha-ketoglutarate |  |
| *Wakabayashi et al 2025[70] | Fibrosis | | Japan | | Support Vector  Machine | 463 | 370 | 93 | NR | Age, sex, BMI, diabetes mellitus, hypertension, hyperlipidemia, AST, ALT, GGT, HbA1c, TG, HDL-C, LDL-C, FBG. |  |
| *Xiong et al 2025[71] | Fibrosis | | China | | XGBoost | 746 | 522 | NR | 224 (Internal validation) | TG, ALB, INR, HDL |  |
| *Calès et al 2025[72] | Fibrosis | | France, Switzerland | | ADORE software | 1,051 | 637 | NR | 414 (Internal validation) | Aspartate aminotransferase, alanine aminotransferase, gamma-glutamyltransferase, ALP, bilirubin, albumin, platelets, prothrombin index [%, or international normalized ratio] and urea, all adjusted on age, weight, height and diabetes,hyaluronate and alpha2-macroglobulin, LSM |  |
| Matuszewska et al 2025[73] | Fibrosis | | Poland | | DT | 178 | 178 | NR | NR | Age, sex, diabetes status, hemoglobin, PLT, AST, GGT, albumin, bilirubin, APRI, INR, PT. |  |
| *Jamialahmadi et al 2025[74] | Fibrosis | | Iran | | LR | 512 | 358 | NR | 154 (Internal validation) | Hemoglobin, FBG, Skeletal Muscle Mass, ALT, Triglycerides, AST. |  |
| Zheng et al 2024[75] | All-cause mortality | | USA | | LR | 3,233 | 2.262 | 971 | NR | Age, gender, ethnicity, BMI, waist circumference, SBP, DBP, WBC, PLT, CRP, iron, lipids, HbA1c, liver enzymes (ALT, AST, GGT, ALP), albumin, bilirubin |  |
| Uehara et al 2018[76] | MASH | | Japan | | Continuous Re-RX with J48graft | 102 | 102 | NR | NR | ALT, CRP, homeostasis model assessment insulin resistance, albumin | |

Note: *refers to studies with available data for final analysis. #Studies did not specify the exact machine learning algorithms used; these cases are labeled as ML in the table. **^△^** indicated that if studies employed various models, only diagnostic metrics of the best-performing models within this study were displayed in this table. Abbreviations: CHAID, chi-squared automatic interaction detector; EML, Ensemble Machine learning; GB, Gradient Boosting; GBM, Gradient Boosting Machine; GMLVQ, Generalized Matrix Learning Vector Quantization; KNN, K-Nearest Neighbor; LR, Logistic Regression; ML, Machine learning; NR, not reported; RF, Random forests; XGBoost, eXtreme Gradient Boosting; ALT, alanine aminotransferase; AST, aspartate aminotransferase; GGT, gamma-glutamyl transferase; HbA1c, glycated hemoglobin; PLT, platelet count; LSM, liver stiffness measurement; CAP, controlled attenuation parameter; VCTE, vibration-controlled transient elastography; AKP, alkaline phosphatase; DBP, diastolic blood pressure; SBP, systolic blood pressure; TC, total cholesterol; TG, total triglyceride; BMI, body mass index; HDL-C, high-density lipoprotein cholesterol; LDL-C, low-density lipoprotein cholesterol; SWE, shear wave elastography; CRP, C-reactive protein; INR, International normalized ratio; PT, Prothrombin time; APRI, aspartateaminotransferase-to-platelet ratio index; ALB, albumin; PDFF, proton density fat fraction; MRE, magnetic resonance elastography; DT, Decision Tree; FBG, fasting blood glucose; APTT, ativated partial thromboplastin time; HOMA, Homeostasis model assessment; UDC, Unsupervised Deep Clustering.

**Table S3. Eligible 30 studies on application of deep learning models.**

| **Study** | **Outcomes** | **Study country/area** | **Algorithm^△^** | **Participants**  **(No.)** | **Training size (No.)** | **Testing size (No.)** | **Validation size (No.)** | **Used features/**  **Supplementary information** |
| --- | --- | --- | --- | --- | --- | --- | --- | --- |
| *Cunha et al 2022[77] | Fibrosis | USA | CNN | 756 | 675 | 81 | NR | CNN-based MRE stiffness measurements |
| Pournik et al 2014[78] | Fibrosis | Iran. | ANN | 396 | 396 | NR | NR | Age, PLT count, albumin, AST/ALT ratio, AST, diabetes, BMI |
| Sherman et al 2024[79] | Fibrosis | USA | DL# | 3,134 | 3,134 | NR | NR | Female, age at biopsy, race/ethnicity, ALT, AST, total bilirubin, PT-INR, BMI, diabetes, dyslipidemia, hypertension |
| Naik et al 2023[80] | Fibrosis | NR | DL# | 152 | 152 | NR | NR | / |
| Zhan et al 2023[81] | Fibrosis | China | AutoFibroNet | 278 | 203 | NR | 75 (Internal validation) | / |
| McNeil et al 2023[82] | MASH | NR | DL# | 192 | 192 | NR | NR | Virtual histologic stains by autofluorescence |
| Naoumov et al 2022[83] | MASH | NR | AI# | 99 | 99 | NR | NR | / |
| *Marti-Aguado et al 2021[84] | MASH | Spain | MATLAB software | 156 | 156 | NR | NR | / |
| Bosch et al 2021[85] | MASH | NR | CNN | 218 | NR | NR | NR | Nodularity, Enhanced Liver Fibrosis, PLT, AST, and bilirubin |
| *Gao et al 2023[86] | MASH | China | FCNN | 261 | 209 | NR | 52 (Internal validation) | Surface enhanced Raman spectroscopy |
| Leow et al 2020[87] | MASH | China | qFibrosis | 160 | 160 | NR | NR | Periportal and perisinusoidal fibrosis characteristics |
| *Li et al 2023[88] | MASH | China | DNN | 766 | 613 | NR | 153 (Internal validation) | Age, sex, prior hypertension, prior diabetes, six body composition (namely arm circumference, percent body fat, bone mineral content, basal metabolic rate, body cell mass and visceral fat area) |
| *Okanoue et al 2021[89] | MASH | Japan | NN | 398 | 324 | NR | 74 (External validation) | Age, sex, height, weight, waist circumference, AST, ALT, gGGT, cholesterol, TG, and PLT |
| *Okanoue et al 2023[90] | MASH | Japan | NN | 434 | 324 | NR | 110 (External validation) | Age, sex, height, weight, waist circumference, AST, ALT, GGT, cholesterol, triglyceride, platelet count, and type 4 collagen 7s |
| Okanoue et al 2023[91] | MASH | Japan | NN | 230 | 175 | NR | 55 (Internal validation) | Age, sex, height, weight, AST, ALT, GGT, cholesterol level, triglyceride level, platelet count, diabetes status, and IgM-free apoptosis inhibitor of macrophage level |
| Pollack et al 2021[92] | MASH | USA | CNN | 149 | 115 | NR | 30 (Internal validation) | Four image sequences (precontrast T1-weighted liver acquisition with volume acquisition water and liver acquisition with volume acquisition fat, 120-second–delay T1-weighted liver acquisition with volume acquisition water, and single-shot fast spin-echo T2 weighted) and clinical data |
| Wagner et al 2023[93] | MASH | NR | NN | 84 | 84 | NR | NR | Fatty acid-binding protein-4 and matrix metalloproteinase-9 |
| Yamaguchi et al 2022[94] | Fibrosis | NR | NN | 898 | 898 | NR | 300 (Internal validation) | Age, sex, height, weight, PLT count, and the levels of AST, ALT, GGT, cholesterol, TG, and type IV collagen 7S. |
| Cheruvu et al 2023[95] | MASH | NR | DL# | ~1000 | ~1000 | NR | NR | MT-ND3, HLA-B, APOC-1,  and APOL-1 (gene) |
| Preechathammawong et al 2024[96] | Fibrosis | Thailand | SMART AI-PATHO | 146 | 146 | NR | NR | BMI, AST, ALT, FIB-4, steatosis percentage, fibrosis percentage |
| Ratziu et al 2024[97] | MASH | France, Belgium, USA, Denmark, Portugal, UK, Greece | CNN | 251 | 251 | NR | NR | Digitized biopsy slides |
| Dai et al 2024[98] | Fibrosis | China | NN | 946 | 671 | 275 | NR | 64 SWE-derived features, demographic and medical history, anthropometric measurements, laboratory Parameters, LSM, medication use |
| Drozdov et al 2024[99] | All-cause mortality | UK | Transformer Neural Network | 1,468 | 940 | 528 | NR | Age, gender, ethnicity, medications (BNF codes), lab results (e.g., albumin, eGFR, AST), hospitalizations (ICD-9/10 codes), A&E attendances, outpatient visits, vital signs (BMI, blood pressure) |
| Fujii et al 2024[100] | Fibrosis | Japan | U-Net、ResNet-50 | 214 | 128 | 42 | 44 (Internal validation) | Morphological: Angle, Aspect ratio, Area, Circularity, Feret, MinFeret, Minor, etc. |
| Goodman et al 2024[101] | Fibrosis | USA, Singapore | QSepta/qNodule | 35 | 25 | NR | 10 (External validation) | Septa, Nodule |
| Leow et al 2024[102] | Fibrosis | Malaysia | AI # | 350 | NR | NR | 350 (External validation) | Triglycerides, total cholesterol, fasting glucose, total bilirubin, AST, ALT, GGT, age, weight, height, gender |
| Seko et al 2025[103] | Fibrosis/liver-related events | Japan | NN | 796 | 796 | NR | NR | 11 clinically assessed parameters from NASH‐Scope8 (age, sex, height, weight, waist circumference, platelet count, and the levels of AST, ALT, GGT, cholesterol, and triglycerides) plus type IV collagen 7S |
| *Chattopadhyay et al 2025[104] | MASH | China | CNN | 137 | 110 | 27 | NR | Ultrasound B-scan image features |
| Matholi et al 2025[105] | MASH | Egypt | MultiLayer Perceptron | 400 | 400 | NR | NR | EP300 mRNA, CPN60 mRNA, AST, D. bilirubin, Albumin, GGT, HbA1c, HOMA-IR, and BMI |
| Abdurrachim et al 2024[106] | Fibrosis | Singapore, the United Kingdom, the USA, etc. | Histolndex AI DP platform | 120 | 120 | NR | NR | Second harmonic generation images, qFibrosis continuous values, qFibrosis stage |

Note: *refers to studies with available data for final analysis. #Studies did not specify the exact deep learning or hybrid artificial intelligence algorithms used; these cases are labeled as DL or AL in the table. **^△^** indicated that if studies employed various models, only diagnostic metrics of the best-performing models within this study were displayed in this table. Abbreviations: A/G, albumin/globulin; ALT, alanine transferase; ALP, alkaline phosphatase; ANN, Artificial Neural Network; AST, aspartate transferase; BMI, body mass index; CAE, Cnvolutional Auto Encoder; CNN, Convolutional Neural Networks; Cre, creatinine; CRP, C-reactive protein; DBP, diastolic blood pressure; DL, deep learning; DNN, Deep Neural Network; GAN, Generative Adversarial Networks; GGT, gamma glutaryl transferase; GPT, glutamic pyruvic transaminase; HA, hyaluronic acid; HbA1c, glycated hemoglobin; HDL-C, high-density lipoprotein cholesterol; LDL, low-density lipoprotein cholesterol; Lp(a), lipoprotein(a); NN, Neural Network; PLT, platelets; PT/INR, prothrombin time testing/International normalized ratio; T2DM, type 2 diabetes mellitus; Tbil, Total bilirubin; TC, total cholesterol; TG, triglyceride; UA, uric acid; WC, waist circumference; WBC, white blood count; AST, aspartate aminotransferase; AFP, alpha fetoprotein; LSM, liver stiffness measurement; SWE, shear wave elastography; eGFR, estimated glomerular filtration rate; INR, International normalized ratio; HOMA, Homeostasis model assessment; FCNN, Full Connect Neural Network.

**Table S4. Metrics of machine learning and/or deep learning-based models for diagnosing MASH.**

| **Study** | **Cohort** | **Outcome** | **Models employed^△^** | **AUROC**  **(95% CI)** | **Accuracy (%)** | **Specificity/TNR (%)** | **Sensitivity/Recall (%)** | **PPV (%)** | **NPV (%)** |
| --- | --- | --- | --- | --- | --- | --- | --- | --- | --- |
| *Bastati et al 2023[27] | Training | MASH | RF-based on UDC | 0.85 (0.76-0.98) | 85.2 | 72.2 | 89.2 | 83.3 | 81.3 |
|  | Internal validation |  |  | NR | 86.7 | 100 | 76.5 | 100 | 76.5 |
| Ben-Assuli et al 2022[2] | Training | MASH | Time-series clustering ML models | NR | 83.6 | NR | NR | NR | NR |
| *Canbay et al 2019[3] | Training | MASH | LR | 0.73 (0.66-0.81) | NR | NR | NR | NR | NR |
| Fialoke et al 2018[5] | Training | MASH | XGB | NR | 79.7 | NR | NR | NR | NR |
| Ghadiri et al 2022[6] | Internal validation | MASH | KNN | NR | 79 | NR | 92 | NR | NR |
| Naderi Yaghouti et al 2024[28] | Training | MASH | RF | NR | 81.32 | 70.49 | 86.04 | NR | NR |
| Sydor et al 2022[29] | Training | MASH vs. Control | ML# | NR | 99.98 | NR | NR | NR | NR |
|  |  | HCC vs. MASH |  | NR | 86.4 | NR | NR | NR | NR |
|  |  | HCC vs. MASH vs. Control |  | NR | 86 | NR | NR | NR | NR |
| Uehara et al 2018[76] | Training | MASH | DL# | NR | NR | 41.7 | 86.2 | 88.9 | 35.7 |
| *Wu et al 2022[45] | Training | MASH | SVM | 0.82 (0.81-0.82) | NR | NR | NR | NR | NR |
| Yilmaz et al 2012[31] | Training | MASH | SVM | NR | 92.5 | 87 | 97.8 | 87.8 | 98.2 |
|  | Testing | MASH |  | NR | 89.6 | 84.1 | 92.5 | 86.4 | 93.2 |
| Ji et al 2022[34] | Training | MASH | RF and LR | NR | 72 | NR | NR | NR | NR |
| *Marti-Aguado et al 2021[84] | Training | MASH | MATLAB software | 0.75 (0.65-0.84) | NR | NR | NR | NR | NR |
| Barbois et al 2021[36] | Training | MASH | CART | NR | NR | 84 | 77 | 68 | 89 |
| Cheruvu et al 2023[95] | Training | MASH | DL# | NR | 60.15 | NR | NR | NR | NR |
|  | Testing | MASH |  | NR | 67.5 | NR | NR | NR | NR |
| *Gao et al 2023[86] | Training | MASH | FCNN | 0.83 (0.70-0.92) | NR | NR | NR | NR | NR |
| *Li et al 2023[88] | Training | MASH | DNN | 0.81 (0.77-0.84) | NR | NR | NR | NR | NR |
| Mamandipoor et al 2023[46] | Training | MASH | XGBoost | NR | NR | NR | NR | 0.8 | 0.78 |
| Masarone et al 2021[40] | Training | MAFL vs (MASH & cirrhosis) | ML# | NR | 94 | NR | 94.1 | 97 | 94 |
|  |  | MASH vs cirrhosis |  | NR | 81.3 | 83.3 | 80 | 88.9 | 71.4 |
| *Okanoue et al 2021[89] | Training | MASH | NN | 0.96 (0.94-0.98) | 81.3 | 83.3 | 80 | 88.9 | 71.4 |
| Pollack et al 2021[92] | Training | MASH | CNN | NR | 78 | 85 | 80 | NR | NR |
| Teramoto et al 2020[42] | Training | MASH | LR | NR | 93.9/90.0 | 1/90.9 | 87.9/90.9 | NR | NR |
| *Njei et al 2024[47] | Training | MASH | XGBoost | 0.95 (0.91-0.97) | NR | 97 | 77 | 61 | 99 |
|  | Testing | MASH |  | 0.94 | 89 | NR | NR | NR | NR |
| Baser et al 2024[49] | Training | MASH | RF | 0.83 | NR | NR | NR | NR | NR |
| Charu et al 2024[50] | External validation | Significant fibrosis-FLINT | Superlearner | 0.79 (0.73-0.84) | NR | NR | NR | NR | NR |
|  |  | Significant fibrosis-NHANES |  | 0.74 (0.68-0.79) | NR | NR | NR | NR | NR |
| Chen et al 2024[51] | Training | MASH | Boruta analysis | 0.915 | NR | NR | NR | NR | NR |
| Drozdov et al 2024[53] | Training | MASH | Gaussian Naive Bayes | 0.72 | NR | NR | NR | 78 | 69 |
| *Feng et al 2024[54] | External validation | Significant fibrosis | LR | 0.753 (0.673-0.834) | NR | NR | NR | NR | NR |
| *Mouskeftara et al 2024[56] | Training | MASH | XGBoost | 0.9 (0.897-0.901) | 86.5 | 80 | 100 | 70.6 | 100 |
| Carr et al 2023[35] | Training | Fibrosis | RF | 0.87 (0.77-0.96) | 79.3 | NR | NR | NR | NR |
|  | Testing | MASH |  | 0.81(0.68-0.9) | 78.2 | NR | NR | NR | NR |
|  | Internal | MASH |  | 0.87 (0.77-0.96) | 79.3 | NR | NR | NR | NR |
| *Zamanian et al 2024[59] | Training | MASH | RF | 0.8253 | 81.51 | 61.75 | 89.49 | NR | NR |
| *Chattopadhyay et al 2025[104] | Training | MASH | ResNet50 | 0.96 | 93 | 92 | 95 | NR | NR |
|  | Testing | MASH |  | 0.92 | 84 | 87 | 81 | NR | NR |
| *Yan et al 2024[60] | Internal validation | MASH | XGBoost | 0.93 | 80 | 83 | 92 | 93 | 70 |
|  | External | MASH |  | 0.9 (0.88-0.93) | 81 | 90 | 78 | 92 | 75 |
| Chen et al 2025[61] | Training | MASH | GP | 0.97 | 92 | 88 | 96 | 89 | 95 |
|  | Internal | MASH |  | 0.79 | 74 | 73 | 74 | 55 | 87 |
| *Huang et al 2025[62] | Training | MASH - Chinese cohort | RF | 0.88 (0.83-0.93) | NR | NR | NR | NR | NR |
|  | Training | MASH - Chinese cohort + Finnish cohort |  | 0.87 (0.83-0.91) | NR | NR | NR | 90.8 | 77.2 |
|  | Testing | MASH - Chinese cohort |  | 0.85 (0.75-0.94) | NR | NR | NR | NR | NR |
|  | Exterrnal validation | MASH - Chinese cohort + Finnish cohort |  | 0.81 (0.75,0.88) | NR | NR | NR | 25.3 | 99.2 |
| Matholi et al 2025[64] | Training | MASH | RF | NR | 100 | NR | 100 | NR | NR |
| *Liu et al 2025[63] | Training | MASH | XGBoost | 0.968 (0.953-0.983) | 91.2 | 89.4 | 93.4 | 88.3 | 94 |
|  | Exterrnal validation |  |  | 0.67 (0.53-0.811) | 47.6 | 40 | 93.3 | 20.6 | 97.3 |
| Matholi et al 2025[105] | Training | MASH | XGBoost | 0.98 | NR | NR | NR | NR | NR |
| Mounika et al 2025[65] | Training | Control vs MASH | RF | 0.98 | 90.98 | 93.76 | 88.2 | NR | NR |
|  |  | MAFL vs MASH |  | 0.9 | 81.58 | 82.3 | 80.86 | NR | NR |
| *Panagiotopoulos et al 2025[68] | Training | MASH | LR | 0.85 (0.79-0.92) | NR | 66 | 77 | NR | NR |

Note: *refers to studies with available data for final analysis. **^△^** indicated that if studies employed various models, only diagnostic metrics of the best-performing models within this study were displayed in this table. #Studies did not specify the exact machine learning or deep learning algorithms used; these cases are labeled as ML or DL in this table. Abbreviations: AUROC, area under the receiver operator characteristic curve; HCC, hepatocellular carcinoma; MAFL, metabolic dysfunction-associated fatty liver; MASH, metabolic dysfunction-associated steatohepatitis; NASH, nonalcoholic steatohepatitis; ML, Machine learning; DL, deep learning; NR, not reported. TNR, true negative rate; PPV, positive predictive value; NPV, negative predictive value; XGBoost, eXtreme Gradient Boosting; RF, Random Forest; LR, Logistic Regression; GP, Gaussian process; FLINT, Farnesoid X nuclear receptor ligand obeticholic acid for non-cirrhotic NASH; NHANES, National Health and Nutrition Examination Survey; UDC, Unsupervised Deep Clustering; SVM, Support Vector Machines; FCNN, Full Connect Neural Network; DNN, Deep Neural Networks; KNN, K-Nearest Neighbor; CART, Classification And Regression Tree; CNN, Convolutional Neural Network.

**Table S5. Metrics of machine learning and/or deep learning-based models for diagnosing liver fibrosis.**

| **Study** | **Cohort** | **Outcome** | **Models**  **employed^△^** | **AUROC**  **(95% CI)** | **Accuracy (%)** | **Specificity/TNR (%)** | **Sensitivity/Recall (%)** | **PPV (%)** | **NPV (%)** |
| --- | --- | --- | --- | --- | --- | --- | --- | --- | --- |
| Yamaguchi et al 2022[94] | Training & Internal validation | F3-4 vs F0-2 (MASH with gray zone)† | NN | NR | NR | 99.6/94.8 | 99.8/98.6 | 99.8/98.1 | 99.6/96.1 |
|  |  | F3-4 vs F0-2 (MASH without gray zone)† |  | NR | NR | 98.7/92.6 | 99.5/98.6 | 99.5/97.3 | 98.7/96.2 |
| Anushiravani et al 2023[1] | Exterrnal validation | Cirrhosis | RF | NR | NR | 62.9 | 70.5 | 15 | 95.8 |
|  |  | ≥F2 |  | NR | NR | 16.4 | 87 | 24.8 | 80 |
| Ben-Assuli et al 2022[2] | Training | Fibrosis | Time-series clustering ML models | NR | 83.6 | NR | NR | NR | NR |
| *Chang et al 2023[43] | Training | MASH with significant fibrosis | RF | 0.80 (0.78-0.83) | 74 | 73 | 78 | 47 | 92 |
|  |  | ≥F2 |  | 0.86 (0.84-0.87) | 79 | 81 | 76 | 75 | 82 |
|  |  | ≥F3 |  | 0.89 (0.87-0.91) | 83 | 87 | 73 | 70 | 89 |
|  |  | Cirrhosis |  | 0.89 (0.86-0.92) | 89 | 94 | 59 | 63 | 93 |
| *Eslam et al 2016[4] | Training | ≥F2 | CART | 0.79 (0.75-0.83) | NR | NR | NR | NR | NR |
|  |  | ≥F3 |  | 0.81 (0.77-0.86) | NR | NR | NR | NR | NR |
|  |  | Cirrhosis |  | 0.84 (0.80-0.87) | NR | NR | NR | NR | NR |
| Pournik et al 2014[78] | Training | Fibrosis | ANN | NR | NR | 99 | 66 | 92 | 93 |
| Schattenberg et al 2023[9] | Training | Fibrosis | ML# | NR | 75 | NR | 81 | 80 | 65 |
| Sokolowska et al 2022[10] | Training | Fibrosis | ML# | NR | 93 | NR | NR | NR | NR |
| *Verma et al 2024[11] | Testing | ≥F2 | RF | 0.72 (0.69-0.75) | NR | 67 | NR | NR | 79.9 |
|  | Internal validation |  |  | NR | NR | 76.6 | NR | NR | 85.2 |
| *Sowa et al 2013[12] | Training | Fibrosis | RF | 0.67 (0.66-0.68) | NR | 77 | 60 | NR | NR |
| *Wu et al 2022[45] | Training | Fibrosis | GB | 0.84 (0.83-0.85) | NR | NR | NR | NR |  |
| Hassoun et al 2024[14] | Training &Testing | Fibrosis | SVM | NR | 78 | NR | 80 | NR | NR |
|  | Exterrnal validation | Fibrosis |  | NR | 69 | 71 | 68 | NR | NR |
| *Chen et al 2021[16] | Training | Fibrosis | LR | 0.82 (0.60-0.95) | NR | NR | NR | NR | NR |
| *Fan et al 2024[17] | Training | ≥F3 | LR | 0.88 (0.78-0.97) | 88.9 | 97.4 | 96.6 | 72.2 | 99.1 |
|  |  | Cirrhosis |  | 0.93 (0.82-1.00) | 82.4 | 92.6 | 91.2 | 63.6 | 97.1 |
| *Feng et al 2021[18] | Training | Fibrosis | ML# | 0.90 (0.87-0.90) | NR | NR | NR | NR | NR |
| Goldman et al 2021[19] | Training | Fibrosis | DT | NR | 85.73 | NR | NR | NR | NR |
| Meng et al 2023[20] | Training | Fibrosis | SVM | NR | NR | NR | 94.9 | NR | NR |
| *Moolla et al 2020[21] | Training | ≥F3 | GMLVQ | 0.99 (0.98-0.99) | NR | NR | NR | NR | NR |
|  |  | Cirrhosis |  | 1.00 (1.00-1.00) | NR | NR | NR | NR | NR |
| Naik et al 2023[80] | Training | Fibrosis | ML+DL# | NR | 78.89 | NR | NR | NR | NR |
| *Okanoue et al 2021[90] | Training | Fibrosis | NN | NR | 99.5 | 90.9 | NR | 97.4 | 98 |
| Sripongpun et al 2022[22] | Training | Fibrosis | LR | NR | NR | NR | 90.7 | NR | 83.6 |
|  | Testing | Fibrosis |  | NR | NR | NR | 97.6/95.7 | NR | 87.5/91.7 |
| Suárez et al 2023[23] | Training | Fibrosis | XGBoost | NR | 93.16 | 93.06 | 93.25 | NR | NR |
| Suárez et al 2023[24] | Training | Fibrosis | Adaboost | NR | 93.53 | 93.71 | 93.32 | NR | NR |
| Suárez et al 2023[25] | Training | Fibrosis | XGBoost | NR | 95.08 | NR | 95.11 | NR | NR |
| *Wang et al 2019[26] | Training & Internal validation | ≥F2 | SVM | 0.95 (0.92-0.98) | NR | 90.8/91.4 | 95.1/78.9 | 88.6/76.8 | 96.1/92.3 |
|  |  | ≥F3 |  | 0.93 (0.89-0.97) | NR | 89.2/95.1 | 88.3/83 | 77.9/72.2 | 94.7/97.3 |
|  |  | Cirrhosis |  | 0.96 (0.93-0.99) | NR | 91.3/96 | 93.9/91.7 | 67.4/72.7 | 98.7/99 |
| *Dabbah et al 2025[52] | Internal validation | F3 | XGBoost (threshold >0.14) | 0.91 (0.88-0.97) | NR | 76 | 91 | 31 | 99 |
|  |  |  | XGBoost (threshold >0.19) | 0.91 (0.88-0.97) | NR | 85 | 87 | 38 | 99 |
| Dai et al[98] | Testing | Fibrosis | DL# | 0.884 | 79.6 | NR | 74.8 | 81.4 | NR |
| Drozdov et al 2024[53] | Training | F3 | Bagged Trees | 0.850 | NR | 64 | 90 | 69 | 88 |
| Fujii et al 2024[100] | Training & Internal validation | ≥F3 | U-Net、ResNet-50 | 0.722 | NR | 86.2 | 57.1 | NR | NR |
|  |  | Cirrhosis |  | 0.825 | NR | 60 | 100 | NR | NR |
| Goodman et al 2024[101] | Training | Cirrhosis | qSepta | 0.91 | NR | NR | 91 | 85 | NR |
|  |  | MASH | qNodule | 0.82 | NR | NR | 82 | 95 | NR |
|  | Internal validation | Cirrhosis | qSepta | NR | NR | NR | 91 | 94 | NR |
|  |  | MASH | qNodule | NR | NR | NR | 94 | 95 | NR |
| Leow et al 2024[102] | External validation | F3 | AI# | 0.79 (0.74-0.85) | NR | 50 | 90 | 30 | 95 |
| Lu et al 2024[55] | Training & Internal validation | F2 | RF | 0.805 | NR | NR | NR | NR | NR |
| Blomdahl et al 2025[57] | Training | F0-1 vs F2-4 | RF | NR | NR | 91.7 | 66.7 | 84 | 80 |
|  | Internal validation |  |  | NR | NR | NR | NR | NR | NR |
|  | External validation |  |  | NR | 70.8 | 70.1 | NR | 68 | 79 |
| *Snethlage et al 2024[58] | Training  & Internal | Fibrosis | Extra Trees | 0.73 (0.727-0.732) | NR | NR | NR | NR | NR |
| *Alkhouri et al 2025[66] | Training | ALADDIN-F2-VCTE (with VCTE data) | RF + GBM + XGBoost | 0.824 (0.801-0.859) | NR | NR | NR | NR | NR |
|  |  | ALADDIN-F2-Lab (without VCTE data) |  | 0.781 (0.753-0.809) | NR | NR | NR | NR | NR |
|  | Testing | ALADDIN-F2-VCTE (with VCTE data) |  | 0.792 (0.768-0.8) | 90.1 | NR | 93.1 | 86.4 | 79.1 |
|  |  | ALADDIN-F2-Lab (without VCTE data) |  | 0.779 (0.756-0.8) | 87.3 | NR | 92.5 | 82.7 | 79.2 |
|  | External validation | ALADDIN-F2-VCTE (with VCTE data) |  | 0.791 (0.764-0.819) | 90.3 | NR | 91.8 | 87.4 | 75.7 |
|  |  | ALADDIN-F2-Lab (without VCTE data) |  | 0.717 (0.69-0.744) | 83.3 | NR | 87.1 | 79.2 | 66.9 |
| Boullion et al 2025[67] | Testing | Fibrosis | RF | NR | 86.07 | 20.92 | 98.01 | 87.12 | 65.79 |
| *Panagiotopoulos et al 2025[68] | Training | Fibrosis | LR | 0.82 (0.71-0.94) | NR | 85 | 67 | NR | NR |
| *Stefanakis et al 2025[69] | Training | Fibrosis | CatBoost | 0.96 (0.95-0.97) | 95 | 95 | 92 | NR | NR |
|  | Internal validation | Fibrosis |  | 0.87 (0.84-0.89) | 87 | 77 | 93 | 63.1 | 92.7 |
| *Wakabayashi et al 2025[70] | Testing | ≥F2 | SVM | 0.886 (0.795-0.941) | NR | 78.5 | 85.7 | 44.7 | 98.2 |
|  |  | ≥F3 |  | 0.882 | NR | 72 | 94.4 | 37.5 | 100 |
|  |  | Cirrhosis |  | 0.916 | NR | 88.5 | 100 | NR | NR |
| Xiong et al 2025[71] | Training | ≥F3 | XGBoost | 0.934 (0.914-0.955 | 86.2 | 74.4 | 95.8 | 85.6 | 87.6 |
|  | Testing |  |  |  |  | 65.8 | 95.9 | 83.7 | 89.7 |
|  | Internal validation |  |  | 0.917(0.88-0.953) | 85.3 | 65.8 | 95.9 | 83.7 | 89.7 |
| *Calès et al 2025[72] | Training | F3 | FIB-12-based on ADORE software | 0.909 | NR | NR | NR | NR | NR |
| Ginter-Matuszewska et al 2025[73] | Training | Fibrosis | DT | 0.728 | 68.4 | 73.8 | 77.1 | NR | NR |
| *Jamialahmadi et al 2025[74] | Internal validation | Fibrosis | LR | 0.72 (0.6-0.8) | 69 | 83 | 51 | 72 | 67 |

Note: *refers to studies with available data for final analysis. #Studies did not specify the exact machine learning or deep learning or artificial intelligence algorithms used; these cases are labeled as ML or DL or AL in the table. **^△^** indicated that if studies employed various models, only diagnostic metrics of the best-performing models within this study were displayed in this table. †MASH patients with gray zone refers to the score of NN-based model within the threshold of 0.167-0.809, and MASH patients without gray zone refers to scores out of the threshold of 0.167-0.809 indicated by Yamaguchi et al[75]. ≥F2 and ≥F3 refers to significant fibrosis and advanced fibrosis, respectively. Abbreviations: AUROC, area under the receiver operator characteristic curve; NR, not reported. DT, Decision Tree; DL, Deep Learning; GB, Gradient Boosting; ANN, Artificial Neural Networks; NN, Neural Network; AdaBoost, Adaboost Adaptive Boosting; GMLVQ, Generalised Matrix Learning Vector Quantisation;;TNR, True Negative Rate; PPV, positive predictive value; NPV, negative predictive value; XGBoost, eXtreme Gradient Boosting; RF, Random forests; LR, Logistic Regression; GP, Gaussian process; SVM, Support Vector Machines.

**Figure S1. Distribution of studies applying machine learning and/or deep learning by the year of publication.**

**
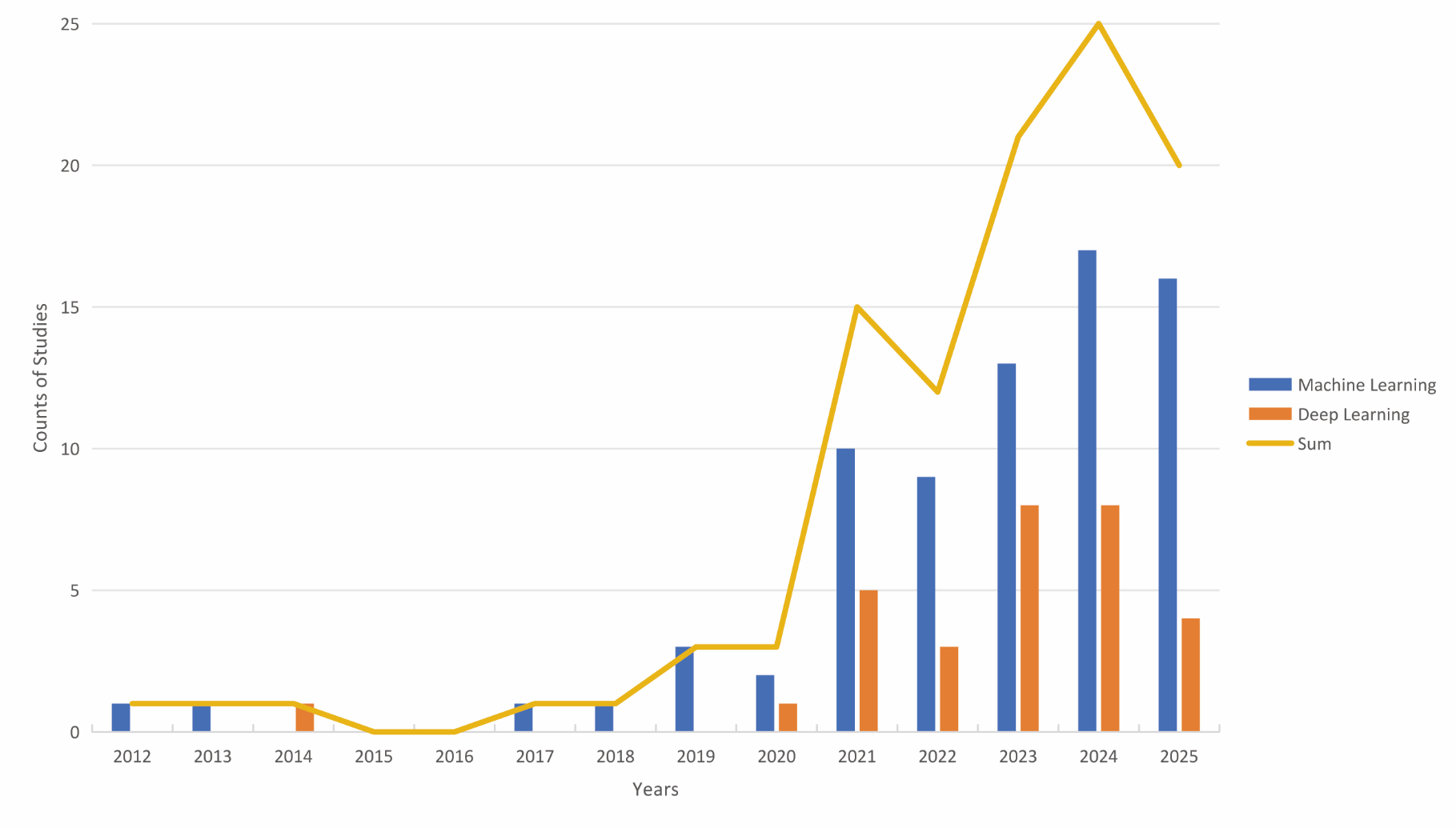
**

**Figure S2. Quality assessment of 35 eligible studies by Quality Assessment of Diagnostic Accuracy Studies tool 2 (QUADAS-2).**


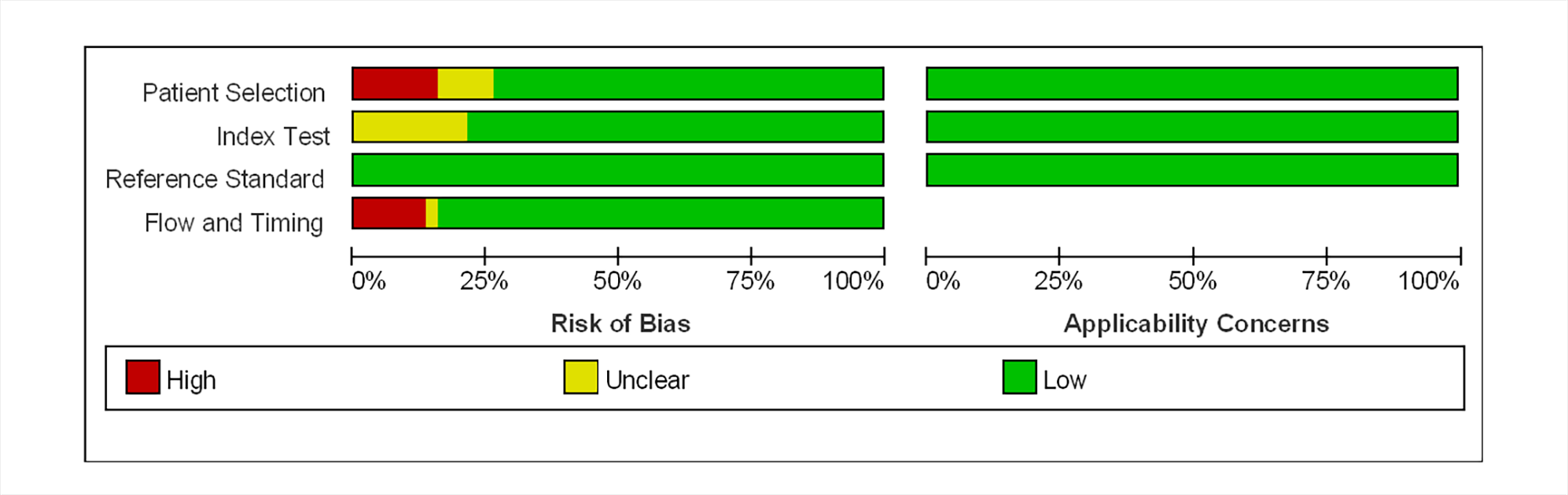

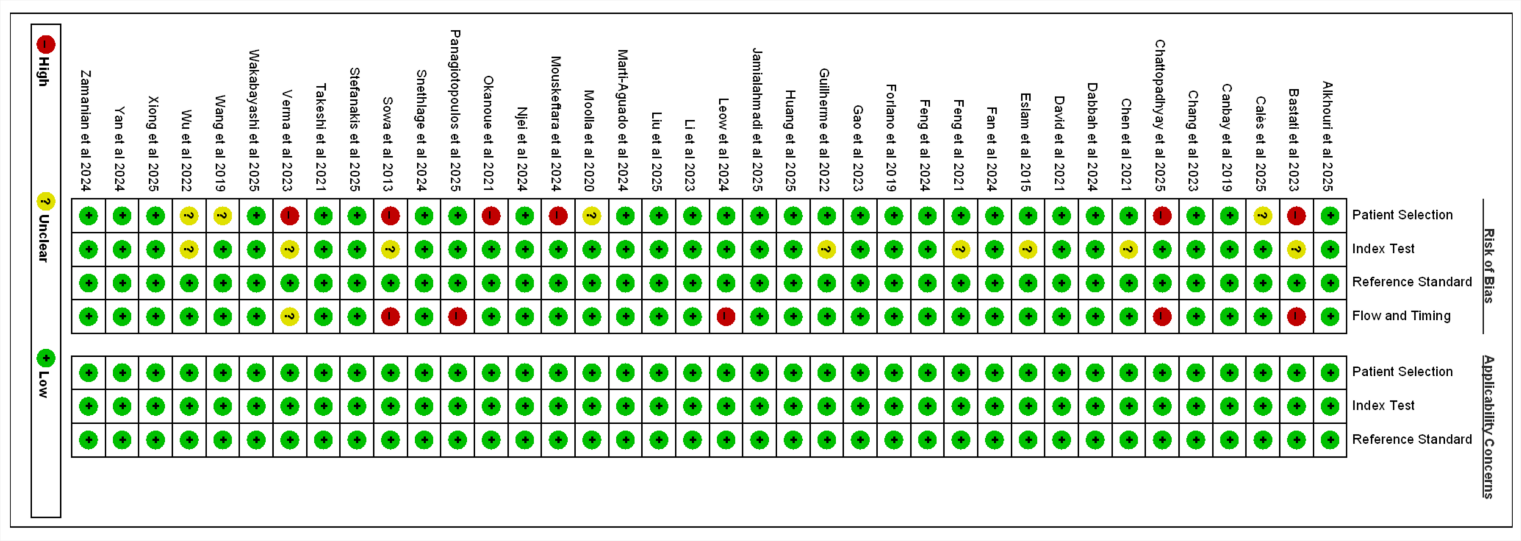


**Figure S3. Forest plots of AUROCs for diagnosing MASH in machine learning and deep learning models.** Abbreviations: AUROC, area under the receiver operator characteristic curve; CI: confidence interval; MASH, metabolic dysfunction-associated steatohepatitis.

**
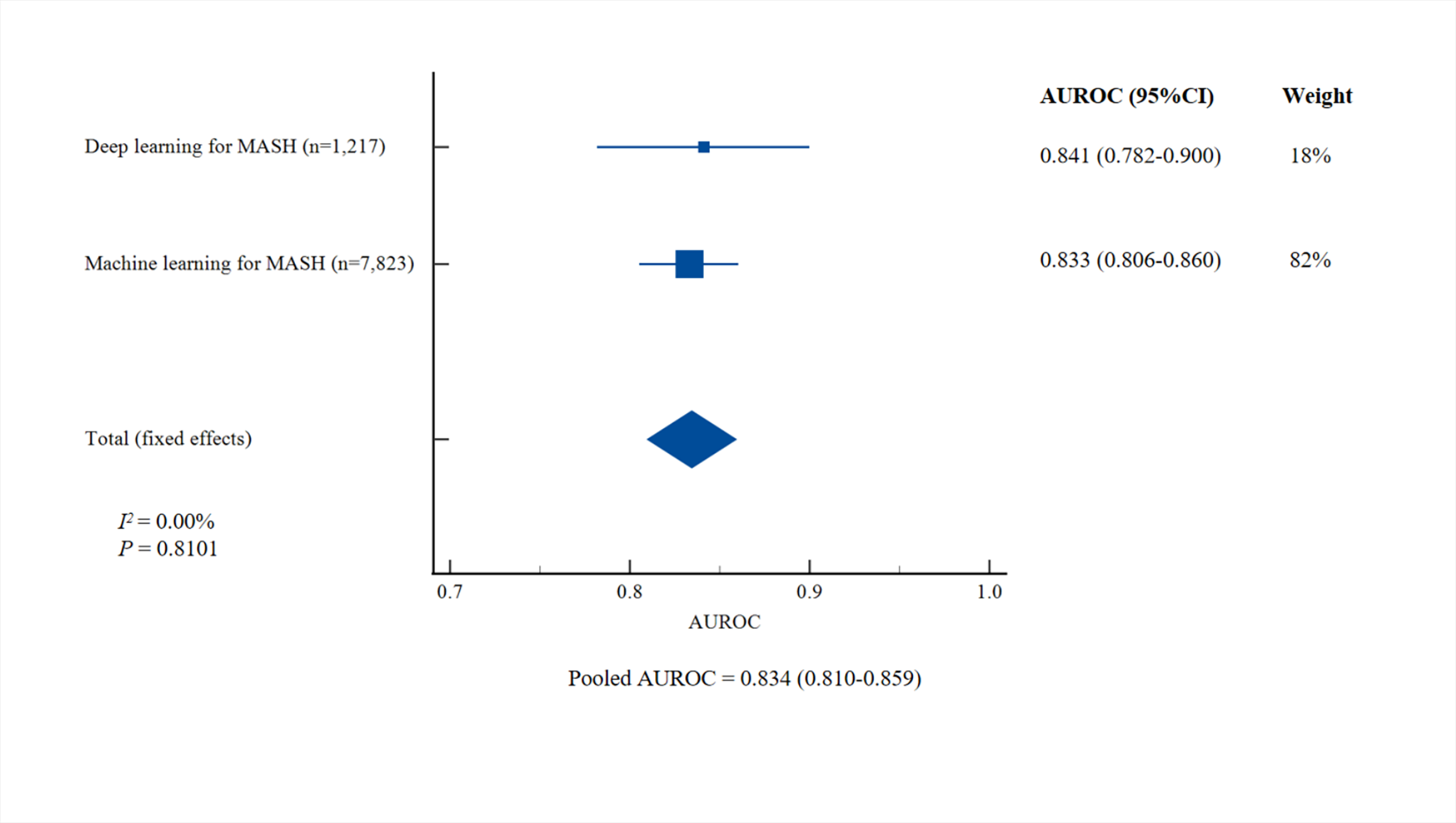
**

**Figure S4. Forest plots of AUROCs for diagnosing liver fibrosis in machine learning and deep learning models.** Abbreviations: AUROC, area under the receiver operator characteristic curve; CI: confidence interval.


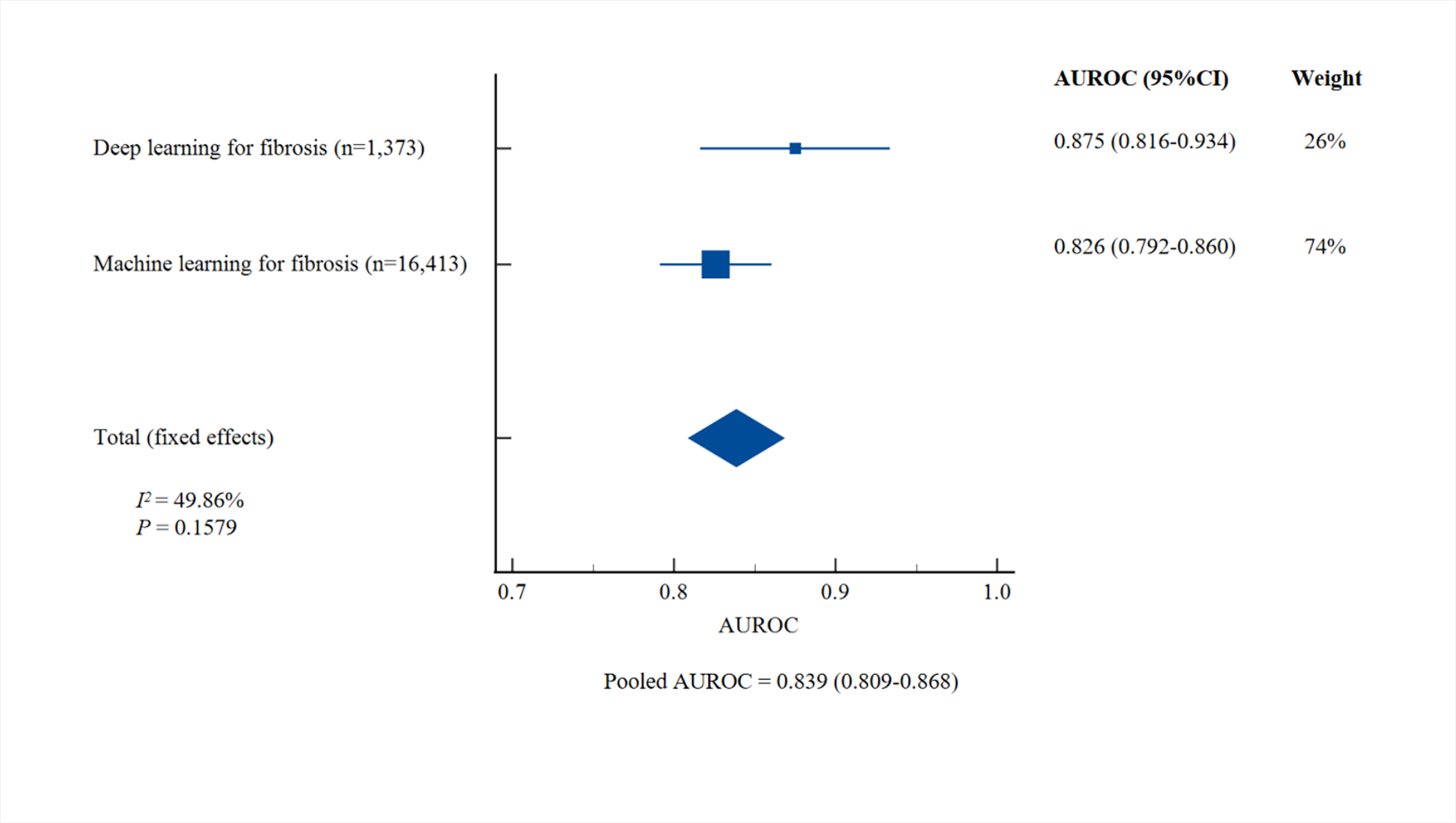


**Figure S5. Forest plots of AUROCs for diagnosing MASH based on machine learning models across different geographic regions. (A) Asia studies; (B) Non-Asia studies.** Abbreviations: AUROC, area under the receiver operator characteristic curve; CI: confidence interval; MASH, metabolic dysfunction-associated steatohepatitis. AdaBoost, Adaboost Adaptive Boosting; BIC, Bayesian Information Criterion; DenseNet, Densely Connected Convolutional Network; DNN, Deep Neural Networks; DT, Decision Tree; FCNN, Fully Connected Neural Network; GB, Gradient Boosting; GBM, Gradient Boosting Machine; KNN, K-Nearest Neighbor; LDA, Linear Discriminant Analysis; LR, Logistic Regression; MLP, Multilayer Perceptron; ResNET, Residual Neural Network; RF, Random forests; SVM, Support Vector Machines; UDC, Unsupervised Deep Clustering; XGBoost, eXtreme Gradient Boosting.


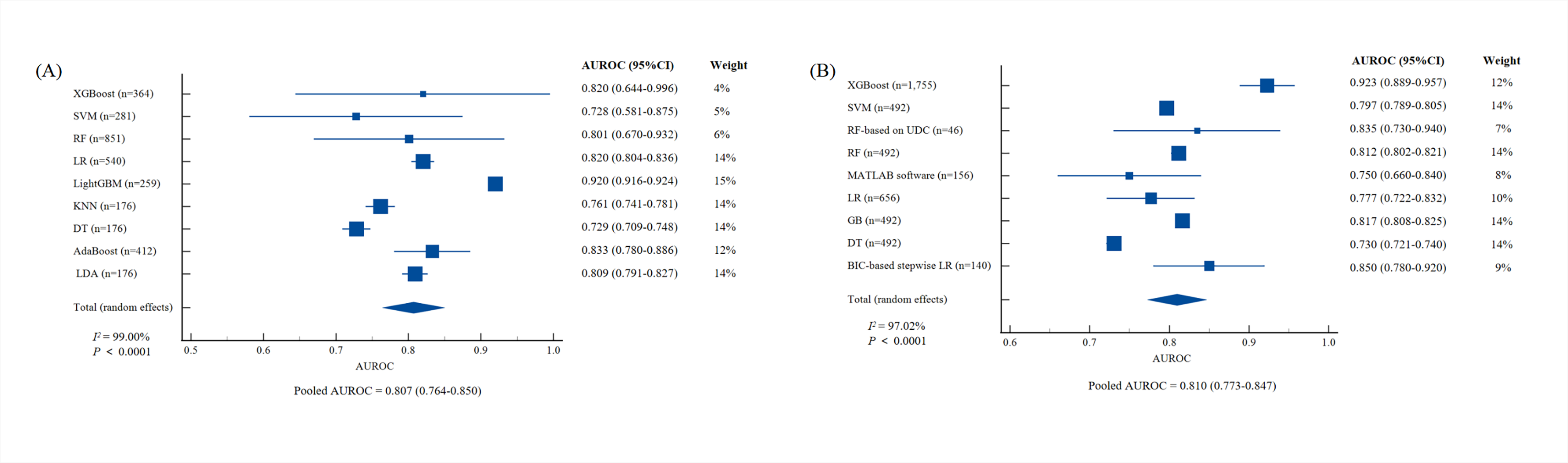


**Figure S6. Forest plots of AUROCs for diagnosing liver fibrosis based on machine learning models across different geographic regions. (A) Asia studies; (B) Non-Asia studies.** Abbreviations: AUROC, area under the receiver operator characteristic curve; CI: confidence interval. ANN, Artificial Neural Network; CART, Classification And Regression Tree; CatBoost, Categorical Gradient Boosting Machines; CNN, Convolutional Neural Network; GB, Gradient Boosting; GBM, Gradient Boosting Machine; GMLVQ, Generalised Matrix Learning Vector Quantisation; LDA, Linear Discriminant Analysis; NN, Neural Network; LR, Logistic Regression; NB, Naive Bayes; RF, Random forests; SVM, Support Vector Machines; XGBoost, eXtreme Gradient Boosting; 5-NN, 5-Nearest Neighbor.


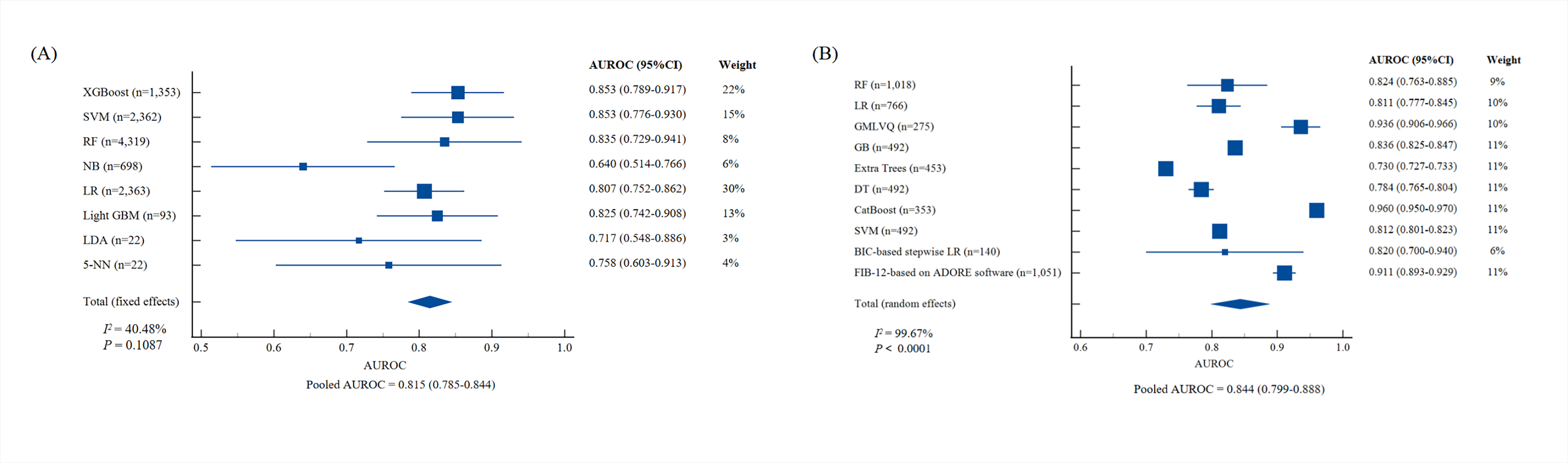


**Figure S7. Forest plots of AUROCs for diagnosing MASH and its related fibrosis based on machine learning models classified by ensemble and non-ensemble methods. (A) MASH diagnosis based on ensemble methods; (B) MASH diagnosis based on non-ensemble methods; (C) Significant fibrosis based on ensemble methods; (D) Significant fibrosis based on non-ensemble methods; (E) Advanced fibrosis based on ensemble methods; (F) Advanced fibrosis based on non-ensemble methods; (G) Cirrhosis based on ensemble methods; (H) Cirrhosis based on non-ensemble methods.** Abbreviations: AUROC, area under the receiver operator characteristic curve; CI: confidence interval; MASH, metabolic dysfunction-associated steatohepatitis. AdaBoost, Adaboost Adaptive Boosting; BIC, Bayesian Information Criterion; CART, Classification And Regression Tree; CatBoost, Categorical Boosting; DT, Decision Tree; FIB-12, Fibrosis-12 index; GB Gradient Boosting; GBM, Gradient Boosting Machine; KNN, K-Nearest Neighbor; LDA, Linear Discriminant Analysis; LR, Logistic Regression; NB, Naive Bayes; RF, Random forests; SVM, Support Vector Machines; UDC, Unsupervised Deep Clustering; XGBoost, eXtreme Gradient Boosting.


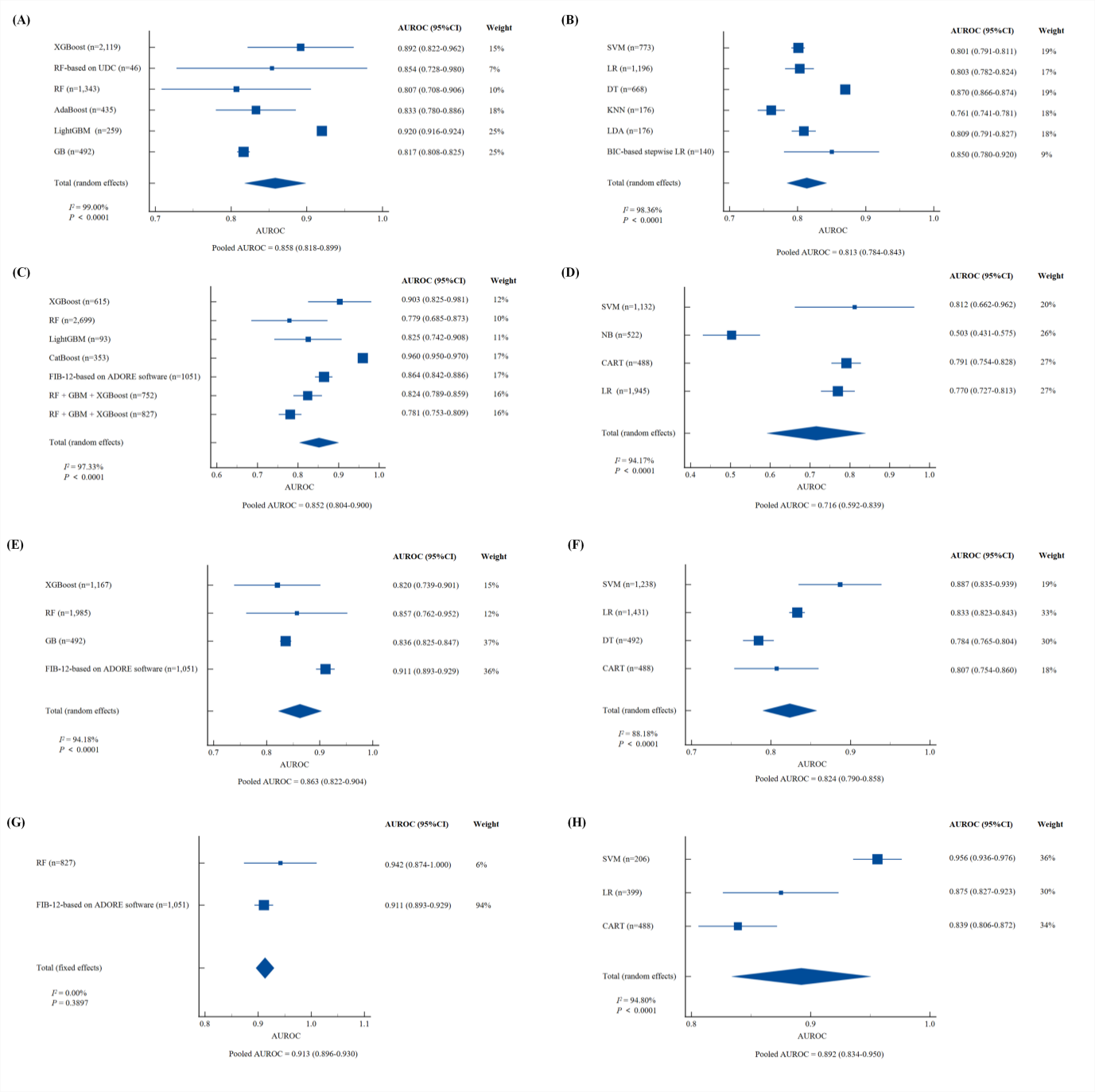


**Figure S8. Forest plots of AUROCs for diagnosing MASH and its related fibrosis based on machine learning models by the year of publications. (A) Studies published within 2019-2022 for diagnosing MASH; (B) Studies published within 2023-2025 for diagnosing MASH; (C) Studies published within 2015-2022 for diagnosing significant fibrosis; (D) Studies published within 2023-2025 for diagnosing significant fibrosis; (E) Studies published within 2013-2022 for diagnosing advanced fibrosis; (F) Studies published within 2023-2025 for diagnosing advanced fibrosis; (G) Studies published within 2015-2022 for diagnosing cirrhosis; (H) Studies published within 2023-2025 for diagnosing cirrhosis.** Abbreviations: AUROC, area under the receiver operator characteristic curve; CI: confidence interval; MASH, metabolic dysfunction-associated steatohepatitis. AdaBoost, Adaboost Adaptive Boosting; BIC, Bayesian Information Criterion; CART, Classification And Regression Tree; CatBoost, Categorical Boosting; DT, Decision Tree; FIB-12, Fibrosis-12 index; GB Gradient Boosting; GBM, Gradient Boosting Machine; KNN, K-Nearest Neighbor; LDA, Linear Discriminant Analysis; LR, Logistic Regression; NB, Naive Bayes; RF, Random forests; SVM, Support Vector Machines; UDC, Unsupervised Deep Clustering; XGBoost, eXtreme Gradient Boosting.


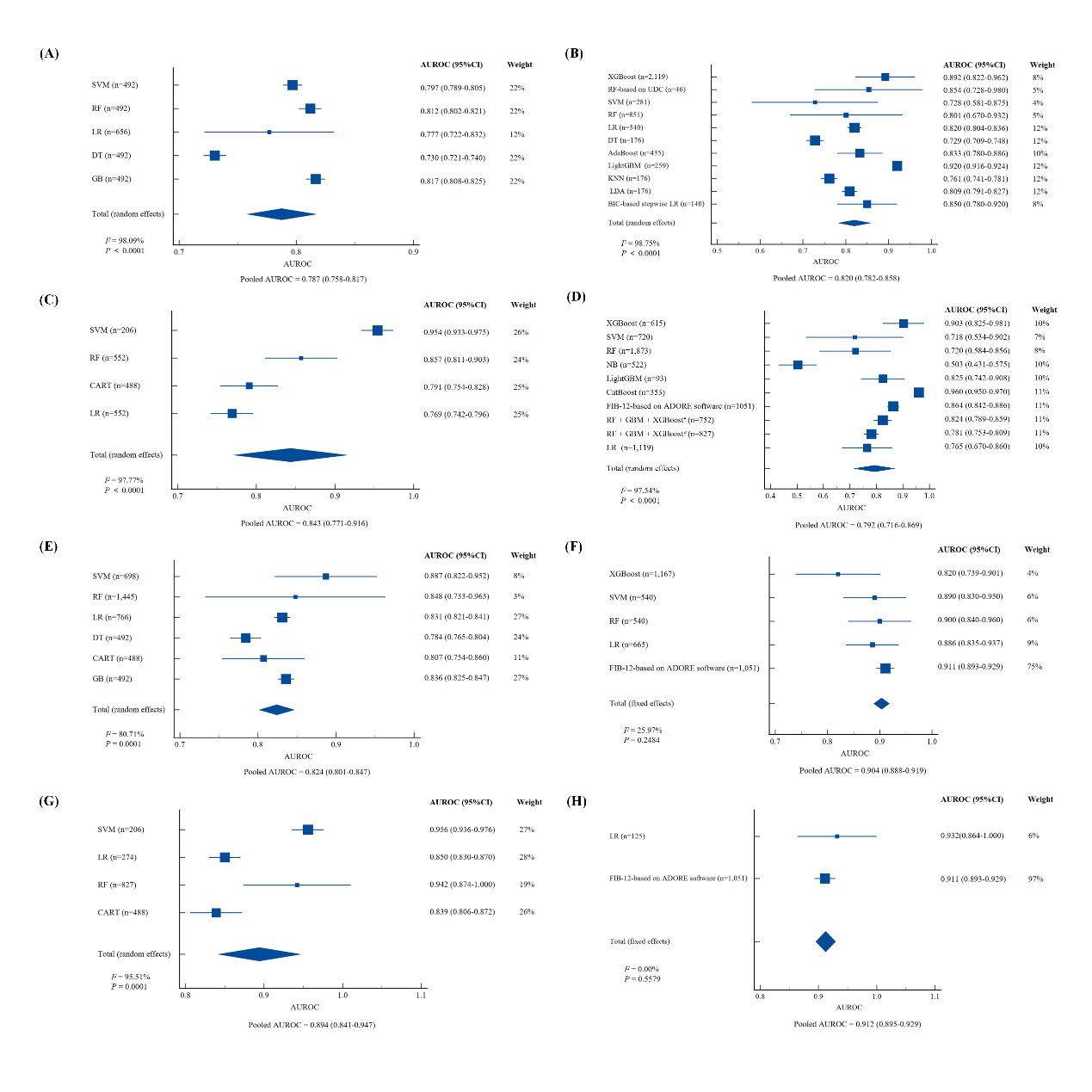


References

[1] A. Anushiravani, K. Alswat, G. N. Dalekos, K. Zachou, N. Örmeci, S. Al-Busafi *et al.*, Multicenter validation of FIB-6 as a novel machine learning non-invasive score to rule out liver cirrhosis in biopsy-proven MAFLD, Eur J Gastroenterol Hepatol*,* 2023; 35(11): 1284-1288.

[2] O. Ben-Assuli, A. Jacobi, O. Goldman, S. Shenhar-Tsarfaty, O. Rogowski, D. Zeltser *et al.*, Stratifying individuals into non-alcoholic fatty liver disease risk levels using time series machine learning models, J Biomed Inform*,* 2022; 126: 103986.

[3] A. Canbay, J. Kälsch, U. Neumann, M. Rau, S. Hohenester, H. A. Baba *et al.*, Non-invasive assessment of NAFLD as systemic disease-A machine learning perspective, PLoS One*,* 2019; 14(3): e0214436.

[4] M. Eslam, A. M. Hashem, M. Romero-Gomez, T. Berg, G. J. Dore, A. Mangia *et al.*, FibroGENE: A gene-based model for staging liver fibrosis, J Hepatol*,* 2016; 64(2): 390-398.

[5] S. Fialoke, A. Malarstig, M. R. Miller, and A. Dumitriu, Application of Machine Learning Methods to Predict Non-Alcoholic Steatohepatitis (NASH) in Non-Alcoholic Fatty Liver (NAFL) Patients, AMIA Annu Symp Proc*,* 2018; 2018: 430-439.

[6] F. Ghadiri, A. A. Husseini, and O. Öztaş, A machine-learning approach for nonalcoholic steatohepatitis susceptibility estimation, Indian J Gastroenterol*,* 2022; 41(5): 475-482.

[7] J. Lee, M. Westphal, Y. Vali, J. Boursier, S. Petta, R. Ostroff *et al.*, Machine learning algorithm improves the detection of NASH (NAS-based) and at-risk NASH: A development and validation study, Hepatology*,* 2023; 78(1): 258-271.

[8] R. Loomba, V. Seguritan, W. Li, T. Long, N. Klitgord, A. Bhatt *et al.*, Gut Microbiome-Based Metagenomic Signature for Non-invasive Detection of Advanced Fibrosis in Human Nonalcoholic Fatty Liver Disease, Cell Metab*,* 2017; 25(5): 1054-1062.e5.

[9] J. M. Schattenberg, M. M. Balp, B. Reinhart, A. Tietz, S. A. Regnier, G. Capkun *et al.*, NASHmap: clinical utility of a machine learning model to identify patients at risk of NASH in real-world settings, Sci Rep*,* 2023; 13(1): 5573.

[10] K. E. Sokolowska, D. Maciejewska-Markiewicz, J. Bińkowski, J. Palma, O. Taryma-Leśniak, K. Kozlowska-Petriczko *et al.*, Identified in blood diet-related methylation changes stratify liver biopsies of NAFLD patients according to fibrosis grade, Clin Epigenetics*,* 2022; 14(1): 157.

[11] N. Verma, A. Duseja, M. Mehta, A. De, H. Lin, V. W. Wong *et al.*, Machine learning improves the prediction of significant fibrosis in Asian patients with metabolic dysfunction-associated steatotic liver disease - The Gut and Obesity in Asia (GO-ASIA) Study, Aliment Pharmacol Ther*,* 2024; 59(6): 774-788.

[12] J. P. Sowa, D. Heider, L. P. Bechmann, G. Gerken, D. Hoffmann, and A. Canbay, Novel algorithm for non-invasive assessment of fibrosis in NAFLD, PLoS One*,* 2013; 8(4): e62439.

[13] V. Charu, J. W. Liang, A. Mannalithara, A. Kwong, L. Tian, and W. R. Kim, Benchmarking clinical risk prediction algorithms with ensemble machine learning: An illustration of the superlearner algorithm for the non-invasive diagnosis of liver fibrosis in non-alcoholic fatty liver disease, medRxiv*,* 2023.

[14] S. Hassoun, C. Bruckmann, S. Ciardullo, G. Perseghin, F. Marra, A. Curto *et al.*, NAIF: A novel artificial intelligence-based tool for accurate diagnosis of stage F3/F4 liver fibrosis in the general adult population, validated with three external datasets, Int J Med Inform*,* 2024; 185: 105373.

[15] L. Brausch, S. Tretbar, H. Hewener, and Ieee, "Identification of advanced hepatic steatosis and fibrosis using ML algorithms on high-frequency ultrasound data in patients with non-alcoholic fatty liver disease," 2021.

[16] Z. W. Chen, K. Tang, Y. F. Zhao, Y. Z. Chen, L. J. Tang, G. Li *et al.*, Radiomics based on fluoro-deoxyglucose positron emission tomography predicts liver fibrosis in biopsy-proven MAFLD: a pilot study, Int J Med Sci*,* 2021; 18(16): 3624-3630.

[17] R. Fan, N. Yu, G. Li, T. Arshad, W. Y. Liu, G. L. Wong *et al.*, Machine-learning model comprising five clinical indices and liver stiffness measurement can accurately identify MASLD-related liver fibrosis, Liver Int*,* 2024; 44(3): 749-759.

[18] G. Feng, K. I. Zheng, Y. Y. Li, R. S. Rios, P. W. Zhu, X. Y. Pan *et al.*, Machine learning algorithm outperforms fibrosis markers in predicting significant fibrosis in biopsy-confirmed NAFLD, J Hepatobiliary Pancreat Sci*,* 2021; 28(7): 593-603.

[19] O. Goldman, O. Ben-Assuli, O. Rogowski, D. Zeltser, I. Shapira, S. Berliner *et al.*, Non-alcoholic Fatty Liver and Liver Fibrosis Predictive Analytics: Risk Prediction and Machine Learning Techniques for Improved Preventive Medicine, J Med Syst*,* 2021; 45(2): 22.

[20] F. Meng, Q. Wu, W. Zhang, and S. Hou, Application of Interpretable Machine Learning Models Based on Ultrasonic Radiomics for Predicting the Risk of Fibrosis Progression in Diabetic Patients with Nonalcoholic Fatty Liver Disease, Diabetes Metab Syndr Obes*,* 2023; 16: 3901-3913.

[21] A. Moolla, J. de Boer, D. Pavlov, A. Amin, A. Taylor, L. Gilligan *et al.*, Accurate non-invasive diagnosis and staging of non-alcoholic fatty liver disease using the urinary steroid metabolome, Aliment Pharmacol Ther*,* 2020; 51(11): 1188-1197.

[22] P. Sripongpun, W. R. Kim, A. Mannalithara, V. Charu, A. Vidovszky, S. Asch *et al.*, The steatosis-associated fibrosis estimator (SAFE) score: A tool to detect low-risk NAFLD in primary care, Hepatology*,* 2023; 77(1): 256-267.

[23] M. Suárez, R. Martínez, A. M. Torres, A. Ramón, P. Blasco, and J. Mateo, A Machine Learning-Based Method for Detecting Liver Fibrosis, Diagnostics (Basel)*,* 2023; 13(18).

[24] M. Suárez, R. Martínez, A. M. Torres, A. Ramón, P. Blasco, and J. Mateo, Personalized Risk Assessment of Hepatic Fibrosis after Cholecystectomy in Metabolic-Associated Steatotic Liver Disease: A Machine Learning Approach, J Clin Med*,* 2023; 12(20).

[25] M. Suárez, R. Martínez, A. M. Torres, B. Torres, and J. Mateo, A Machine Learning Method to Identify the Risk Factors for Liver Fibrosis Progression in Nonalcoholic Steatohepatitis, Dig Dis Sci*,* 2023; 68(9): 3801-3809.

[26] Y. Wang, G. L. Wong, F. P. He, J. Sun, A. W. Chan, J. Yang *et al.*, Quantifying and monitoring fibrosis in non-alcoholic fatty liver disease using dual-photon microscopy, Gut*,* 2020; 69(6): 1116-1126.

[27] N. Bastati, M. Perkonigg, D. Sobotka, S. Poetter-Lang, R. Fragner, A. Beer *et al.*, Correlation of histologic, imaging, and artificial intelligence features in NAFLD patients, derived from Gd-EOB-DTPA-enhanced MRI: a proof-of-concept study, Eur Radiol*,* 2023; 33(11): 7729-7743.

[28] A. R. Naderi Yaghouti, H. Zamanian, and A. Shalbaf, Machine learning approaches for early detection of non-alcoholic steatohepatitis based on clinical and blood parameters, Sci Rep*,* 2024; 14(1): 2442.

[29] S. Sydor, C. Dandyk, J. Schwerdt, P. Manka, D. Benndorf, T. Lehmann *et al.*, Discovering Biomarkers for Non-Alcoholic Steatohepatitis Patients with and without Hepatocellular Carcinoma Using Fecal Metaproteomics, Int J Mol Sci*,* 2022; 23(16).

[30] O. Yasar, P. Long, B. Harder, H. Marshall, S. Bhasin, S. Lee *et al.*, Machine learning using longitudinal prescription and medical claims for the detection of non-alcoholic steatohepatitis (NASH), BMJ Health Care Inform*,* 2022; 29(1).

[31] Y. Yilmaz, and F. Eren, Identification of a support vector machine-based biomarker panel with high sensitivity and specificity for nonalcoholic steatohepatitis, Clin Chim Acta*,* 2012; 414: 154-7.

[32] Q. Zhu, H. Li, Z. Ao, H. Xu, J. Luo, C. Kaurich *et al.*, Lipidomic identification of urinary extracellular vesicles for non-alcoholic steatohepatitis diagnosis, J Nanobiotechnology*,* 2022; 20(1): 349.

[33] H. Jiang, Y. Hu, Z. Zhang, X. Chen, and J. Gao, Identification of metabolic biomarkers associated with nonalcoholic fatty liver disease, Lipids Health Dis*,* 2023; 22(1): 150.

[34] M. Ji, Y. Jo, S. J. Choi, S. M. Kim, K. K. Kim, B. C. Oh *et al.*, Plasma Metabolomics and Machine Learning-Driven Novel Diagnostic Signature for Non-Alcoholic Steatohepatitis, Biomedicines*,* 2022; 10(7).

[35] R. M. Carr, Y. Li, L. Chau, E. S. Friedman, J. J. Lee, L. Adorini *et al.*, An integrated analysis of fecal microbiome and metabolomic features distinguish non-cirrhotic NASH from healthy control populations, Hepatology*,* 2023; 78(6): 1843-1857.

[36] S. Barbois, N. Stürm, J. Aron-Wisnewsky, K. Clément, P. Bedossa, L. Genser *et al.*, Decision Tree for the Performance of Intraoperative Liver Biopsy During Bariatric Surgery, Obes Surg*,* 2021; 31(6): 2641-2648.

[37] M. Docherty, S. A. Regnier, G. Capkun, M. M. Balp, Q. Ye, N. Janssens *et al.*, Development of a novel machine learning model to predict presence of nonalcoholic steatohepatitis, J Am Med Inform Assoc*,* 2021; 28(6): 1235-1241.

[38] R. García-Carretero, R. Holgado-Cuadrado, and Ó. Barquero-Pérez, Assessment of Classification Models and Relevant Features on Nonalcoholic Steatohepatitis Using Random Forest, Entropy (Basel)*,* 2021; 23(6).

[39] Y. Luo, S. Wadhawan, A. Greenfield, B. E. Decato, A. M. Oseini, R. Collen *et al.*, SOMAscan Proteomics Identifies Serum Biomarkers Associated With Liver Fibrosis in Patients With NASH, Hepatol Commun*,* 2021; 5(5): 760-773.

[40] M. Masarone, J. Troisi, A. Aglitti, P. Torre, A. Colucci, M. Dallio *et al.*, Untargeted metabolomics as a diagnostic tool in NAFLD: discrimination of steatosis, steatohepatitis and cirrhosis, Metabolomics*,* 2021; 17(2): 12.

[41] M. Noureddin, Z. Goodman, D. Tai, E. L. K. Chng, Y. Ren, P. Boudes *et al.*, Machine learning liver histology scores correlate with portal hypertension assessments in nonalcoholic steatohepatitis cirrhosis, Aliment Pharmacol Ther*,* 2023; 57(4): 409-417.

[42] T. Teramoto, T. Shinohara, and A. Takiyama, Computer-aided classification of hepatocellular ballooning in liver biopsies from patients with NASH using persistent homology, Comput Methods Programs Biomed*,* 2020; 195: 105614.

[43] D. Chang, E. Truong, E. A. Mena, F. Pacheco, M. Wong, M. Guindi *et al.*, Machine learning models are superior to noninvasive tests in identifying clinically significant stages of NAFLD and NAFLD-related cirrhosis, Hepatology*,* 2023; 77(2): 546-557.

[44] N. Perakakis, S. A. Polyzos, A. Yazdani, A. Sala-Vila, J. Kountouras, A. D. Anastasilakis *et al.*, Non-invasive diagnosis of non-alcoholic steatohepatitis and fibrosis with the use of omics and supervised learning: A proof of concept study, Metabolism*,* 2019; 101: 154005.

[45] Y. Wu, X. Yang, H. L. Morris, M. J. Gurka, E. A. Shenkman, K. Cusi *et al.*, Noninvasive Diagnosis of Nonalcoholic Steatohepatitis and Advanced Liver Fibrosis Using Machine Learning Methods: Comparative Study With Existing Quantitative Risk Scores, JMIR Med Inform*,* 2022; 10(6): e36997.

[46] B. Mamandipoor, S. Wernly, G. Semmler, M. Flamm, C. Jung, E. Aigner *et al.*, Machine learning models predict liver steatosis but not liver fibrosis in a prospective cohort study, Clin Res Hepatol Gastroenterol*,* 2023; 47(7): 102181.

[47] B. Njei, E. Osta, N. Njei, Y. A. Al-Ajlouni, and J. K. Lim, An explainable machine learning model for prediction of high-risk nonalcoholic steatohepatitis, SCIENTIFIC REPORTS*,* 2024; 14(1).

[48] S. Sarkar, A. Alurwar, C. Ly, C. Piao, R. Donde, C. J. Wang *et al.*, A Machine Learning Model to Predict Risk for Hepatocellular Carcinoma in Patients With Metabolic Dysfunction-Associated Steatotic Liver Disease, GASTRO HEP ADVANCES*,* 2024; 3(4): 498-505.

[49] O. Baser, G. Samayoa, N. Yapar, and E. Baser, Artificial Intelligence in Identifying Patients With Undiagnosed Nonalcoholic Steatohepatitis, J Health Econ Outcomes Res*,* 2024; 11(2): 86-94.

[50] V. Charu, J. W. Liang, A. Mannalithara, A. Kwong, L. Tian, and W. R. Kim, Benchmarking clinical risk prediction algorithms with ensemble machine learning for the noninvasive diagnosis of liver fibrosis in NAFLD, HEPATOLOGY*,* 2024; 80(5).

[51] J. Chen, R. S. Lu, C. Diaz-Canestro, E. Song, X. Jia, Y. Liu *et al.*, Distinct changes in serum metabolites and lipid species in the onset and progression of NAFLD in Obese Chinese, Comput Struct Biotechnol J*,* 2024; 23: 791-800.

[52] S. Dabbah, I. Mishani, Y. Davidov, and Z. Ben Ari, Implementation of Machine Learning Algorithms to Screen for Advanced Liver Fibrosis in Metabolic Dysfunction-Associated Steatotic Liver Disease: An In-Depth Explanatory Analysis, Digestion*,* 2024: 1-14.

[53] Y. Davidov, R. Y. Brzezinski, M. I. Kaufmann, M. Likhter, T. Hod, O. Pappo *et al.*, Incorporating artificial intelligence in portable infrared thermal imaging for the diagnosis and staging of nonalcoholic fatty liver disease, JOURNAL OF BIOPHOTONICS*,* 2024.

[54] S. S. Feng, S. J. Wang, L. Guo, P. P. Ma, X. L. Ye, M. L. Pan *et al.*, Serum bile acid and unsaturated fatty acid profiles of non-alcoholic fatty liver disease in type 2 diabetic patients, WORLD JOURNAL OF DIABETES*,* 2024; 15(5).

[55] C. H. Lu, W. Wang, Y. C. J. Li, I. W. Chang, C. L. Chen, C. W. Su *et al.*, Machine Learning Models for Predicting Significant Liver Fibrosis in Patients with Severe Obesity and Nonalcoholic Fatty Liver Disease, OBESITY SURGERY*,* 2024; 34(12): 4393-4404.

[56] T. Mouskeftara, G. Kalopitas, T. Liapikos, K. Arvanitakis, G. Germanidis, and H. Gika, Predicting Non-Alcoholic Steatohepatitis: A Lipidomics-Driven Machine Learning Approach, INTERNATIONAL JOURNAL OF MOLECULAR SCIENCES*,* 2024; 25(11).

[57] J. Blomdahl, M. Aberg, M. Fridén, H. Ahlström, P. Hockings, J. Hulthe *et al.*, Proteomic signatures for fibrosis in MASLD: a biopsy-proven dual-cohort study, SCANDINAVIAN JOURNAL OF GASTROENTEROLOGY*,* 2025.

[58] C. M. F. Snethlage, A. S. Meijnikman, A. L. Mak, E. Rampanelli, B. Voermans, C. A. K. Callender *et al.*, Prevalence and predictive features of metabolic dysfunction-associated steatotic liver disease in type 1 diabetes, EUROPEAN JOURNAL OF ENDOCRINOLOGY*,* 2024; 190(5): 391-400.

[59] H. Zamanian, and A. Shalbaf, Estimation of non-alcoholic steatohepatitis (NASH) disease using clinical information based on the optimal combination of intelligent algorithms for feature selection and classification, Comput Methods Biomech Biomed Engin*,* 2024; 27(8): 964-979.

[60] Y. Yan, D. Gan, P. Zhang, H. Zou, and M. Li, A machine learning-based predictive model discriminates nonalcoholic steatohepatitis from nonalcoholic fatty liver disease, Heliyon*,* 2024; 10(21): e38848.

[61] J. Chen, B. Zhang, Y. Cheng, Y. Jia, and B. Zhou, Machine Learning-Based Non-Invasive Prediction of Metabolic Dysfunction-Associated Steatohepatitis in Obese Patients: A Retrospective Study, Diagnostics (Basel)*,* 2025; 15(9).

[62] Q. X. Huang, S. F. Qadri, H. Bian, X. X. Yi, C. H. Lin, X. Y. Yang *et al.*, A metabolome-derived score predicts metabolic dysfunction-associated steatohepatitis and mortality from liver disease, JOURNAL OF HEPATOLOGY*,* 2025; 82(5).

[63] M. Liu, L. Jiang, J. Yang, Y. Yao, X. Puyang, X. Ge *et al.*, Development and Validation of a Machine Learning-based Model for Prediction of Liver Fibrosis and MASH, J Clin Gastroenterol*,* 2025.

[64] M. Matboli, N. E. El-Attar, I. Abdelbaky, R. Khaled, M. Saad, A. M. A. Ghani *et al.*, Unveiling NLR pathway signatures: EP300 and CPN60 markers integrated with clinical data and machine learning for precision NASH diagnosis, CYTOKINE*,* 2025; 188.

[65] N. Mounika, S. B. Mungase, S. Verma, S. Kaur, U. J. Deka, T. S. Ghosh *et al.*, Inflammatory Protein Signatures as Predictive Disease-Specific Markers for Non-Alcoholic Steatohepatitis (NASH), INFLAMMATION*,* 2025; 48(1): 25-41.

[66] N. Alkhouri, T. Cheuk-Fung Yip, L. Castera, M. Takawy, L. A. Adams, N. Verma *et al.*, ALADDIN: A Machine Learning Approach to Enhance the Prediction of Significant Fibrosis or Higher in Metabolic Dysfunction-Associated Steatotic Liver Disease, Am J Gastroenterol*,* 2025.

[67] J. Boullion, A. Husein, A. Agrawal, D. Xing, M. I. Hossain, M. S. Bhuiyan *et al.*, Machine Learning-Based Biomarker Identification for Early Diagnosis of Metabolic Dysfunction-Associated Steatotic Liver Disease, JOURNAL OF CLINICAL ENDOCRINOLOGY & METABOLISM*,* 2025.

[68] N. Panagiotopoulos, T. Wolfson, D. T. Harris, D. Batakis, R. Agni, L. Ceriani *et al.*, Proton density fat fraction for diagnosis of metabolic dysfunction-associated steatotic liver disease, HEPATOLOGY*,* 2025.

[69] K. Stefanakis, G. Mingrone, J. George, and C. S. Mantzoros, Accurate non-invasive detection of MASH with fibrosis F2-F3 using a lightweight machine learning model with minimal clinical and metabolomic variables, METABOLISM-CLINICAL AND EXPERIMENTAL*,* 2025; 163.

[70] S. I. Wakabayashi, T. Kimura, N. Tamaki, T. Iwadare, T. Okumura, H. Kobayashi *et al.*, AI-Based Platelet-Independent Noninvasive Test for Liver Fibrosis in MASLD Patients, JGH OPEN*,* 2025; 9(4).

[71] F. X. Xiong, L. Sun, X. J. Zhang, J. L. Chen, Y. Zhou, X. M. Ji *et al.*, Machine learning-based models for advanced fibrosis in non-alcoholic steatohepatitis patients: A cohort study, WORLD JOURNAL OF GASTROENTEROLOGY*,* 2025; 31(9).

[72] P. Calès, C. M. Canivet, C. Costentin, A. Lannes, F. Oberti, I. Fouchard *et al.*, A new generation of non-invasive tests of liver fibrosis with improved accuracy in MASLD, JOURNAL OF HEPATOLOGY*,* 2025; 82(5).

[73] B. Ginter-Matuszewska, A. Adamek, M. Majchrzak, B. Rozplochowski, A. Zientarska, A. Kowala-Piaskowska *et al.*, FibrAIm - The machine learning approach to identify the early stage of liver fibrosis and steatosis, INTERNATIONAL JOURNAL OF MEDICAL INFORMATICS*,* 2025; 197.

[74] T. Jamialahmadi, M. A. Looha, S. Jangjoo, N. Emami, M. A. Abdalla, M. Ganjali *et al.*, Predictive performance of noninvasive factors for liver fibrosis in severe obesity: a screening based on machine learning models, JOURNAL OF DIABETES AND METABOLIC DISORDERS*,* 2025; 24(1).

[75] J. R. Zheng, Z. L. Wang, and B. Feng, "Machine learning‑based mortality prediction models for non-alcoholic fatty liver disease in the general United States population," (Zheng J.-R.; Wang Z.-L.; Feng B., fengbo@pkuph.edu.cn) Peking University People's Hospital, Peking University Hepatology Institute, Beijing Key Laboratory of Hepatitis C and Immunotherapy for Liver Diseases, Beijing, China, 2024.

[76] D. Uehara, Y. Hayashi, Y. Seki, S. Kakizaki, N. Horiguchi, H. Tojima *et al.*, Non-invasive prediction of non-alcoholic steatohepatitis in Japanese patients with morbid obesity by artificial intelligence using rule extraction technology, World J Hepatol*,* 2018; 10(12): 934-943.

[77] G. M. Cunha, T. I. Delgado, M. S. Middleton, S. Liew, W. C. Henderson, D. Batakis *et al.*, Automated CNN-Based Analysis Versus Manual Analysis for MR Elastography in Nonalcoholic Fatty Liver Disease: Intermethod Agreement and Fibrosis Stage Discriminative Performance, AJR Am J Roentgenol*,* 2022; 219(2): 224-232.

[78] O. Pournik, S. Dorri, H. Zabolinezhad, S. M. Alavian, and S. Eslami, A diagnostic model for cirrhosis in patients with non-alcoholic fatty liver disease: an artificial neural network approach, Med J Islam Repub Iran*,* 2014; 28: 116.

[79] M. S. Sherman, P. K. Challa, E. M. Przybyszewski, R. M. Wilechansky, E. N. Uche-Anya, A. T. Ott *et al.*, A natural language processing algorithm accurately classifies steatotic liver disease pathology to estimate the risk of cirrhosis, Hepatol Commun*,* 2024; 8(4).

[80] S. N. Naik, R. Forlano, P. Manousou, R. Goldin, and E. D. Angelini, Fibrosis severity scoring on Sirius red histology with multiple-instance deep learning, Biol Imaging*,* 2023; 3: e17.

[81] H. Zhan, S. Chen, F. Gao, G. Wang, S. D. Chen, G. Xi *et al.*, AutoFibroNet: A deep learning and multi-photon microscopy-derived automated network for liver fibrosis quantification in MAFLD, Aliment Pharmacol Ther*,* 2023; 58(6): 573-584.

[82] C. McNeil, P. F. Wong, N. Sridhar, Y. Wang, C. Santori, C. H. Wu *et al.*, An End-to-End Platform for Digital Pathology Using Hyperspectral Autofluorescence Microscopy and Deep Learning-Based Virtual Histology, Mod Pathol*,* 2024; 37(2): 100377.

[83] N. V. Naoumov, D. Brees, J. Loeffler, E. Chng, Y. Ren, P. Lopez *et al.*, Digital pathology with artificial intelligence analyses provides greater insights into treatment-induced fibrosis regression in NASH, J Hepatol*,* 2022; 77(5): 1399-1409.

[84] D. Marti-Aguado, M. Fernández-Patón, C. Alfaro-Cervello, C. Mestre-Alagarda, M. Bauza, A. Gallen-Peris *et al.*, Digital Pathology Enables Automated and Quantitative Assessment of Inflammatory Activity in Patients with Chronic Liver Disease, Biomolecules*,* 2021; 11(12).

[85] J. Bosch, C. Chung, O. M. Carrasco-Zevallos, S. A. Harrison, M. F. Abdelmalek, M. L. Shiffman *et al.*, A Machine Learning Approach to Liver Histological Evaluation Predicts Clinically Significant Portal Hypertension in NASH Cirrhosis, Hepatology*,* 2021; 74(6): 3146-3160.

[86] F. Gao, D. C. Lu, T. L. Zheng, S. Geng, J. C. Sha, O. Y. Huang *et al.*, Fully connected neural network-based serum surface-enhanced Raman spectroscopy accurately identifies non-alcoholic steatohepatitis, Hepatol Int*,* 2023; 17(2): 339-349.

[87] W. Q. Leow, P. Bedossa, F. Liu, L. Wei, K. H. Lim, W. K. Wan *et al.*, An Improved qFibrosis Algorithm for Precise Screening and Enrollment into Non-Alcoholic Steatohepatitis (NASH) Clinical Trials, Diagnostics (Basel)*,* 2020; 10(9).

[88] G. Li, T. L. Zheng, X. L. Chi, Y. F. Zhu, J. J. Chen, L. Xu *et al.*, LEARN algorithm: a novel option for predicting non-alcoholic steatohepatitis, Hepatobiliary Surg Nutr*,* 2023; 12(4): 507-522.

[89] T. Okanoue, T. Shima, Y. Mitsumoto, A. Umemura, K. Yamaguchi, Y. Itoh *et al.*, Artificial intelligence/neural network system for the screening of nonalcoholic fatty liver disease and nonalcoholic steatohepatitis, Hepatol Res*,* 2021; 51(5): 554-569.

[90] T. Okanoue, T. Shima, Y. Mitsumoto, A. Umemura, K. Yamaguchi, Y. Itoh *et al.*, Novel artificial intelligent/neural network system for staging of nonalcoholic steatohepatitis, Hepatol Res*,* 2021; 51(10): 1044-1057.

[91] T. Okanoue, K. Yamaguchi, T. Shima, Y. Mitsumoto, T. Katayama, K. Okuda *et al.*, Artificial intelligence/neural network system that accurately diagnoses hepatocellular carcinoma in nonalcoholic steatohepatitis, Hepatol Res*,* 2023; 53(12): 1213-1223.

[92] B. L. Pollack, K. Batmanghelich, S. S. Cai, E. Gordon, S. Wallace, R. Catania *et al.*, Deep Learning Prediction of Voxel-Level Liver Stiffness in Patients with Nonalcoholic Fatty Liver Disease, Radiol Artif Intell*,* 2021; 3(6): e200274.

[93] J. Wagner, Y. Kumar, A. Lautenbach, P. von Kroge, S. Wolter, O. Mann *et al.*, Fatty acid-binding protein-4 (FABP4) and matrix metalloproteinase-9 (MMP9) as predictive values for nonalcoholic steatohepatitis (NASH), Lipids Health Dis*,* 2023; 22(1): 1.

[94] K. Yamaguchi, T. Shima, Y. Mitsumoto, Y. Seko, A. Umemura, Y. Itoh *et al.*, Fibro-Scope V1.0.1: an artificial intelligence/neural network system for staging of nonalcoholic steatohepatitis, Hepatol Int*,* 2023; 17(3): 573-583.

[95] A. Cheruvu, D. Zezulinski, and A. Sayeed, "Application of Attention and Graph Transformer-Based Approaches for RNA Biomarker Discovery in Metabolically-Associated Fatty Liver Disease (MAFL/NASH)," (Cheruvu A., cheruvu.a144@student.cbsd.org) Central Bucks South High School, Warrington, PA, United States, 2023.

[96] N. Preechathammawong, M. Charoenpitakchai, N. Wongsason, J. Karuehardsuwan, T. Prasoppokakorn, P. Pitisuttithum *et al.*, Development of a diagnostic support system for the fibrosis of nonalcoholic fatty liver disease using artificial intelligence and deep learning, KAOHSIUNG JOURNAL OF MEDICAL SCIENCES*,* 2024; 40(8): 757-765.

[97] V. Ratziu, S. Francque, C. A. Behling, V. Cejvanovic, H. Cortez-Pinto, J. S. Iyer *et al.*, Artificial intelligence scoring of liver biopsies in a phase II trial of semaglutide in nonalcoholic steatohepatitis, Hepatology*,* 2024; 80(1): 173-185.

[98] R. H. Dai, M. M. Sun, M. Lu, and L. H. Deng, Deep learning for predicting fibrotic progression risk in diabetic individuals with metabolic dysfunction-associated steatotic liver disease initially free of hepatic fibrosis, HELIYON*,* 2024; 10(13).

[99] I. Drozdov, B. Szubert, I. A. Rowe, T. J. Kendall, and J. A. Fallow, Accurate prediction of all-cause mortality in patients with metabolic dysfunction-associated steatotic liver disease using electronic health records, ANNALS OF HEPATOLOGY*,* 2024; 29(5).

[100] I. Fujii, N. Matsumoto, M. Ogawa, A. Konishi, M. Kaneko, Y. Watanabe *et al.*, Artificial Intelligence and Image Analysis-Assisted Diagnosis for Fibrosis Stage of Metabolic Dysfunction-Associated Steatotic Liver Disease Using Ultrasonography: A Pilot Study, DIAGNOSTICS*,* 2024; 14(22).

[101] Z. Goodman, K. Akbary, M. Noureddin, Y. Ren, E. Chng, D. Tai *et al.*, Enhancing Histology Detection in MASH Cirrhosis for Artificial Intelligence Pathology Platform by Expert Pathologist Training, Liver International Communications*,* 2024; 5(4).

[102] Y. W. Leow, W. L. Chan, L. L. Lai, N. R. N. Mustapha, S. Mahadeva, R. Quiambao *et al.*, LIVERSTAT for risk stratification for patients with metabolic dysfunction-associated fatty liver disease, JOURNAL OF GASTROENTEROLOGY AND HEPATOLOGY*,* 2024; 39(10): 2182-2189.

[103] Y. Seko, K. Yamaguchi, T. Shima, S. Tanaka, T. Shirono, Y. Takahashi *et al.*, Prognostic performance of a two-step method using the Fibro-Scope system for metabolic dysfunction-associated steatotic liver disease, HEPATOLOGY RESEARCH*,* 2025; 55(2): 211-218.

[104] T. Chattopadhyay, C. H. Lu, Y. P. Chao, C. Y. Wang, D. I. Tai, M. W. Lai *et al.*, Ultrasound detection of nonalcoholic steatohepatitis using convolutional neural networks with dual-branch global–local feature fusion architecture, Medical and Biological Engineering and Computing*,* 2025.

[105] M. Matboli, S. Hamady, M. Saad, R. Khaled, A. Khaled, E. M. Barakat *et al.*, Innovative approaches to metabolic dysfunction-associated steatohepatitis diagnosis and stratification, NON-CODING RNA RESEARCH*,* 2025; 10: 206-222.

[106] D. Abdurrachim, S. Lek, C. Z. L. Ong, C. K. Wong, Y. Zhou, A. Wee *et al.*, Utility of AI digital pathology as an aid for pathologists scoring fibrosis in MASH, J Hepatol*,* 2025; 82(5): 898-908.
